# Novel Mitoviruses and a Unique Tymo-Like Virus in Hypovirulent and Virulent Strains of the Fusarium Head Blight Fungus, *Fusarium boothii*

**DOI:** 10.3390/v10110584

**Published:** 2018-10-26

**Authors:** Yukiyoshi Mizutani, Adane Abraham, Kazuma Uesaka, Hideki Kondo, Haruhisa Suga, Nobuhiro Suzuki, Sotaro Chiba

**Affiliations:** 1 Graduate School of Bioagricultural Sciences, Nagoya University, Nagoya 464-8601, Japan; m.yukiyosi@gmail.com; 2 Institute of Plant Science and Resources, Okayama University, Kurashiki 710-0046, Japan; adaneab2016@gmail.com (A.A.); hkondo@okayama-u.ac.jp (H.K.); nsuzuki@okayama-u.ac.jp (N.S.); 3 Center for Gene Research, Nagoya University, Nagoya 464-8601, Japan; uesaka@gene.nagoya-u.ac.jp; 4 Life Science Research Center, Gifu University, Gifu 501-1193, Japan; suga@gifu-u.ac.jp; 5 Asian Satellite Campuses Institute, Nagoya University, Nagoya 464-8601, Japan

**Keywords:** Fusarium head blight, mycovirus, RNA genome, mitovirus, *Tymovirales*, Ethiopia

## Abstract

Hypovirulence of phytopathogenic fungi are often conferred by mycovirus(es) infections and for this reason many mycoviruses have been characterized, contributing to a better understanding of virus diversity. In this study, three strains of Fusarium head blight fungus (*Fusarium boothii*) were isolated from Ethiopian wheats as dsRNA-carrying strains: hypovirulent Ep-BL13 (>10, 3 and 2.5 kbp dsRNAs), and virulent Ep-BL14 and Ep-N28 (3 kbp dsRNA each) strains. The 3 kbp-dsRNAs shared 98% nucleotide identity and have single ORFs encoding a replicase when applied to mitochondrial codon usage. Phylogenetic analysis revealed these were strains of a new species termed Fusarium boothii mitovirus 1 in the genus *Mitovirus*. The largest and smallest dsRNAs in Ep-BL13 appeared to possess single ORFs and the smaller was originated from the larger by removal of its most middle part. The large dsRNA encoded a replicase sharing the highest amino acid identity (35%) with that of Botrytis virus F, the sole member of the family *Gammaflexiviridae.* Given that the phylogenetic placement, large genome size, simple genomic and unusual 3′-terminal RNA structures were far different from members in the order *Tymovirales*, the virus termed Fusarium boothii large flexivirus 1 may form a novel genus and family under the order.

## 1. Introduction

The number of extant fungal species is currently estimated at up to 1.5 million. Viruses that infect fungi are known as mycoviruses or fungal viruses and are ubiquitous across the kingdom Fungi [1]. Partitiviruses, and mitoviruses in particular, are omnipresent. Interestingly, mycoviruses were often found to have unique features such as genomic structures, virion structures, anti-viral defence strategies, etc. that differ from major plant and animal viruses [2,3,4]. Most mycoviruses infect fungal hosts asymptomatically and the remainder rarely exert a positive or negative impact on fungal host traits such as virulence, asexual/sexual spore production, pigmentation and growth [5,6,7]. Virocontrol, a method of biological control using mycoviruses that attenuate the virulence of phytopathogenic fungi (conferring hypovirulence), has attracted the attention of researchers for the last few decades [8,9,10,11], and information on mycovirus diversity has expanded and deepened dramatically through intensive mycovirus hunting aiming at screening for useful virocontrol agents [1]. However, while mycovirus hunting has been extensive in Asian, European, American and Oceanian countries, its exploration in the African continent has been limited: Diaporthe ambigua RNA virus (an unclassified RNA virus), Diplodia scrobiculata RNA virus 1 (a fusagravirus) and Sphaeropsis sapinea RNA virus 1 and 2 (victoriviruses) are reported in South Africa [12,13,14,15,16] with the remainder being mycovirus-like sequences obtained by metagenomic virome approaches [17].

Fusarium head blight (FHB) is an economically important disease of wheat caused by members of the *Fusarium graminearum* species complex (FGSC) that consists of at least 16 species including *F. boothii*. The disease symptoms are reduced yields, shrivelled kernels, reduced seed quality, and diminished seed weight. In addition, the pathogen leaves mycotoxins such as deoxynivalenol (DON) and nivalenol, type B trichothecenes, on grain surface which can cause diarrhoea and vomiting in animals ingesting them. An increasing number of mycoviruses have been reported to infect various *Fusarium* species, including FGSC from different regions of the world. These viruses include members of *Chrysoviridae*, *Hypoviridae*, *Partitiviridae*, *Narnaviridae*, *Totiviridae*, and *Mymonaviridae* families; proposed Alternaviridae and Fusariviridae families; and other unclassified virus groups [9,18,19,20,21,22,23,24,25,26,27]. Those with the greatest potential as virocontrol agents are: Fusarium graminearum virus 1 strain DK21 (FgV1-DK21), Fusarium graminearum virus strain China 9, Fusarium graminearum hypovirus 2 strain JS16, and Fusarium virguliforme dsRNA mycovirus 1 and 2 [18,21,23,28].

As abovementioned, mitoviruses in the genus *Mitovirus* in the family *Narnaviridae* are omnipresent in filamentous fungi and the members have approximately 3 kb positive sense single stranded RNA ((+)ssRNA) genomes that possess the single open reading frame (ORF) encoding an RNA-dependent RNA polymerase (RdRp) [29]. The ORF accommodates “UGA” triplets that is an interrupting stop codon when applied nuclear gene code but translated as tryptophan in the mitochondrial gene code. Cryphonectria parasitica mitovirus 1 (CpMV1) is the first mitovirus described, and co-presence of virus-derived double stranded RNA (dsRNA; considered as a replicative form) with subcellularly fractionated mitochondria suggests the localization of CpMV1 in mitochondria [30]. Khalifa and Pearson [31] observed the accumulation of filamentous structures in mitochondria of *Sclerotinia sclerotiorum* under electron microscope, specifically when a mitovirus infected to the host. Therefore, mitoviruses are expected to uniquely replicate in the host mitochondria. Botrytis cinerea mitovirus 1, Thanatephorus cucumeris mitovirus (TcMV, known also as Rhizoctonia M2 virus), Sclerotinia sclerotiorum mitovirus 1/KL-1, Sclerotinia sclerotiorum mitovirus 2/KL-1, Sclerotinia sclerotiorum mitovirus 3/NZ1, and Sclerotinia sclerotiorum mitovirus 4/NZ1 were shown to have a hypovirulence-conferring ability but effects of most mitoviruses on host fungal phenotypes have not been elucidated because mitoviruses are generally hard to cure and introduce [7,31,32,33,34,35]. Several mitoviruses have also been reported from *Fusarium* species including FGSC but biological properties of those are largely unknown [25,36,37].

The order *Tymovirales* contains viruses that have a non-segmented (+)ssRNA genome with single or multiple ORFs and its members mostly infect plants. The order consists of five families—*Alphaflexiviridae*, *Betaflexiviridae*, *Gammaflexiviridae*, *Deltaflexiviridae* and *Tymoviridae* in International Committee on Taxonomy of Viruses (ICTV)—based on the genome organisation, phylogenetic relationships and virion morphology. As of now, *Alpha*-, *Gamma*- and *Deltaflexiviridae* include mycoviruses and, to our knowledge, at least 10 such mycoviruses have been characterised including the ones associated with hosts that exhibit hypovirulent phenotypes [20,27,38,39,40,41,42,43,44,45].

Botrytis virus F (BVF, *Gammaflexiviridae*) was isolated from a hypovirulent strain of *Botrytis cinerea* as the first mycovirus taxonomically classified in the order *Tymovirales*, and was followed by Botrytis virus X (BVX, *Alphaflexiviridae*) [39,40]. *S. sclerotiorum* also hosts mycoviruses of this order, namely Sclerotinia sclerotiorum debilitation-associated RNA virus (SsDRV, *Alphaflexiviridae*) and Sclerotinia sclerotiorum deltaflexivirus 1 (SsDFV1, *Deltaflexiviridae*). The former is associated with hypovirulence of its host and the latter is recognised as the first mycovirus in the order *Tymovirales* that has no capsid protein (CP) [41,42]. Recently, Rhizoctonia solani flexivirus 1 (RsFV-1, unclassified deltaflexivirus-like) was reported to have the largest genome in the order and its unique genomic organisation broadened our perception of genome plasticity within the order [38]. In addition, two mycoviruses, Fusarium graminearum mycotymovirus 1 (FgMTV1, unclassified tymovirus-like) and Fusarium graminearum deltaflexivirus 1 (FgDFV1, *Deltaflexiviridae*), have been isolated from the FHB pathogen, *F. graminearum* [20,27]. Biological assays demonstrated that FgMTV1 infection does not alter the host virulence, but decreases the growth rate and DON production. Finally, although oyster mushroom spherical virus (OMSV) was earlier reported to be a tymo-like mycovirus [44], its taxonomic placement is inconclusive till date.

This study characterised a novel member of *Tymovirales*, potentially forming a new genus or family, and members of a new species in the genus *Mitovirus* in the family *Narnaviridae*. These virus classes were identified for the first time in *Fusarium boothii* strains from the African continent. It is noteworthy that the tymo-like virus possesses the largest genome size (over 12.5 kb) among the currently reported *Tymovirales* members. This study contributes to our understanding of *Tymovirales* virus and mitovirus diversity and evolution.

## 2. Materials and Methods

### 2.1. Fungal Strains and Culture

*F. boothii* strains (Ep-BL13, Ep-BL14 and Ep-N28) were isolated from wheats with the FHB symptoms in Hadiya region of southern Ethiopia. A Japanese isolate of *F. graminearum* sensu stricto (s.str.) strain was also used as a reference. All strains were maintained at 28 °C on potato dextrose agar (PDA, BD Difco Laboratories, Detroit, MI, USA) and synthetic low nutrient agar (SNA) and stored at 4 °C in the dark. Mycelia were cultured in potato dextrose broth (PDB, BD Difco Laboratories, Detroit, MI, USA) liquid medium at room temperature for 4–7 days for nucleic acid extractions.

### 2.2. Biological Assessment of FGSC Strains

Growth rate and colony morphology. Mycelial plugs were transplanted on PDA and SNA plates (three replicates) and incubated at 20 °C for 1 week. The longest and shortest diameters of each colony were measured 3–7 days post-transplanting and the mean diameter was used for the calculation of colony area.

Pathogenicity test. Conidial spores (1 × 10^5^ spores/mL) suspended in a 1% (*v*/*v*) Triton solution were used for inoculation, and the FHB-sensitive wheat cultivar Apogee was grown at 27 °C in a chamber (KG-50HLA; Koito Electric Industries, Yokohama, Japan) to avoid possible release of inocula into the environment [46,47]. Briefly, 10 µL of spore suspension was injected into wheat spikelets, and the number of diseased spikelets was counted at 15 days post-inoculation. Alternatively, the spore suspension was sprayed three times (500 µL/push) to a wheat head, and disease severity was evaluated according to Ban and Suenaga’s criteria (index, mild to severe; 0, 5, 10, 20, 30, 50, 60, 80, 100) at 15 days post-inoculation [48]. Inoculated plants were placed in a transparent humid box for one day (injection method) or two days (spray method) to promote fungal germination, prior to the maintenance in the chamber.

### 2.3. Genomic Polymerase Chain Reaction

Fungal genomic DNA was purified by conventional CTAB (cetyl trimethylammonium bromide) extraction. The partial internal transcribed spacer (ITS) of rDNA and transcription elongation factor 1-α gene (*TEF1-α*) sequences were PCR-amplified with the oligonucleotide primers reported by O’Donnel et al. [49,50]. Primers used in this study were: primer 1 (5′-TCAAAATGGGTAAGGA(A/G)GACAAGAC-3′) and primer 2 (5′-GCCTGGGA(G/A)GTACCAGT(G/C)ATCATGTT-3′) for ITS; and primer 3 (5′-GTGGGGCATTTACCCCGCC-3′) and primer 4 (5′-GAGTGGCGGGGTAAATGCC-3′) for *TEF1-α*. Fungal species were identified by homology search with and by phylogenetic analysis with sequences from public databases.

### 2.4. dsRNA Extraction and cDNA Library Construction

dsRNA fraction was extracted from fungal mycelia by conventional cellulose column chromatography. Filtrated mycelia were homogenised in the presence of liquid nitrogen, and total RNA fractions were obtained by treatment with one round each of phenol, phenol–chloroform–isoamylalcohol (25:24:1), and chloroform–isoamylalcohol (24:1) extraction. dsRNAs were further isolated from the total RNA fractions by using Cellulose Powder C (Advantec, Tokyo, Japan). To eliminate fungal chromosomal DNA and ssRNA species, these fractions were treated with RNase-free RQ1 DNase I (Promega, Madison, WI, USA) and S1 Nuclease (Promega). Conventional cDNA library construction was performed for viral sequence determination. The dsRNAs extracted from *F. boothii* Ep-BL13, Ep-BL14 and EP-N28 and the 3 kbp- and 2.5-kbp dsRNA bands were gel-purified using a Zymoclean™ Gel RNA Recovery kit (Zymo Research, Irvine, CA, USA). cDNA libraries of each dsRNA fragment were constructed using a non-PCR and a PCR-based method. After denaturation of dsRNA templates at 99 °C for 5 min, cDNA was synthesised using ReverTra Ace-α-(Toyobo, Osaka, Japan) with an adapter-tagged primer (5′-CCTGAATTCGGATCCTCCNNNNNN-3′). The resulting cDNA fragments were purified from an agarose gel with a size exclusion (1.0–2.0 kbp) and amplified by PCR with an adapter primer (5′-CCTGAATTCGGATCCTCC-3′). The PCR conditions are as follows: 94 °C (2 min), then 1 cycle at 94 °C (2 min)/65 °C (1 min)/72 °C (1 min), followed by 35 cycles at 94 °C (40 s)/52 °C (30 s)/72 °C (3 min), and a final extension at 72 °C for 7 min. DNA fragments were cloned into the pGEM-Teasy (Promega) or pCR-Blunt (Thermo Fisher, Waltham, MA, USA) cloning vectors and used for the transformation of *Escherichia coli* strain DH5α for Sanger sequencing analyses.

### 2.5. RNA Seq, Reverse Transcription PCR and Rapid Amplification of cDNA Ends

RNA seq. The *F. boothii* Ep-BL13 virome was established on an Illumina-Hiseq 2500 system (Illumina, San Diego, CA, USA) by a pair-end sequencing run (2 × 100 bp) using 10 ng of dsRNA as a template. cDNA and library construction were outsourced to BGI Japan. Fastqc version 0.11.5 software (https://www.bioinformatics.babraham.ac.uk/projects/fastqc/) was used to check the quality of the read data [51]. Reads were filtered using Sickle version 1.33 software (https://github.com/najoshi/sickle) by requiring an average Phred quality (Q score) of at least 30 and to only retain reads of 20 nucleotides (nt) or greater in length [52]. De novo assembly was carried out using SPAdes version 3.11.1 software (http://cab.spbu.ru/software/spades/) at the default settings [53].

Gap-filling RT-PCR. Target cDNAs were produced using ReverTra Ace and a gene-specific primer set from given dsRNA fragments and amplified with DNA polymerases such as PrimeSTAR (Takara, Shiga, Japan), KOD FX Neo (Toyobo) or GO-Taq (Promega).

3′-RLM-RACE (RNA ligase mediated RACE). The terminal sequences of dsRNAs were determined by this method. Pre-denatured dsRNAs in dimethyl sulphoxide (DMSO) (90%, 65 °C) were ligated at their 3′-ends with a 5′-phosphorylated oligodeoxynucleotide, 3′-RACE-adaptor (5′-CAATACCTTCTGACCATGCAGTGACAGTCAGCATG-3′), using T4 RNA ligase (Takara) at 16 °C for 16 h. Ligated DNA–RNA strands were DMSO-denatured in the presence of the oligonucleotide 3′-RACE-1st (5′-CATGCTGACTGTCACTGCAT-3′) and used as templates for cDNA synthesis. The resulting cDNA was amplified by PCR with 3′-RNACE-2nd (5′-TGCATGGTCAGAAGGTATTG-3′) and gene-specific primers. Plasmid clones and genomic- or RT-PCR amplicons were purified with spin columns (GeneElute, Sigma, St. Louis, MO, USA; Nucleospin, Takara) and used for BigDye (ABI, ThermoFisher) sequencing by following the manufacturer’s instructions. Sanger sequencing was carried out on a 3100-Avant sequencer (ABI/HITACHI). All virus-specific oligonucleotide primers used in this study were designed based on sequences obtained from cDNA library sequencing and Illumina sequencing.

### 2.6. Bioinformatics Analyses

Sequence assemblies were manipulated using GeneStudio (http://genestudio.com/), SPAdes and Genetyx (Genetyx, Tokyo, Japan) software. Sequence homology searches were performed with the Basic Local Alignment Search Tool (BLAST) programme provided by the National Center for Biotechnology Information (NCBI, http://www.ncbi.nlm.nih.gov/) and the Genetyx. Motif searching was conducted using the online tool, InterPro: protein sequence analysis & classification (https://www.ebi.ac.uk/interpro/) [54]. For secondary RNA structure prediction, the 3′- and 5′-terminal viral sequences were submitted to mfold (http://unafold.rna.albany.edu/?q=mfold/rna-folding-form) and potential stem-loops were predicted [55].

### 2.7. Phylogenetic Analysis

Multiple amino acid or nucleotide sequence alignments were constructed using MUSCLE [56] and with the MEGA X [57]. Phylogenetic analysis was conducted by using PhyML ver. 3.0 (http://www.atgc-ontpellier.fr/phyml/) with the best-fit models suggested by Smart Model Selection in PhyML (http://www.atgc-montpellier.fr/sms/) [58], and the results were visualised with FigTree and further enhanced in Adobe Illustrator.

## 3. Results

### 3.1. Detection of dsRNAs in F. boothii Strains

**Figure 1:**
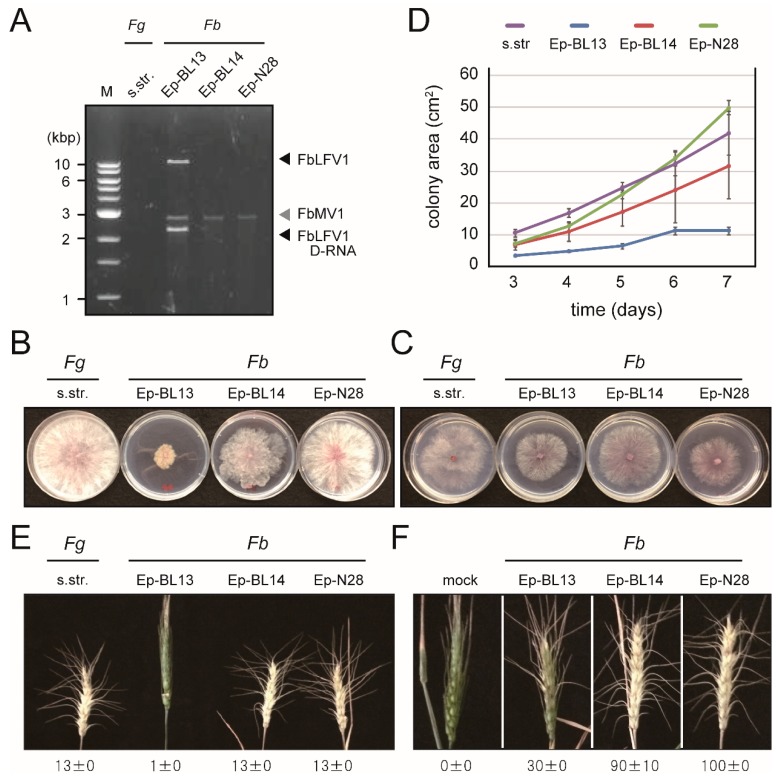
Biological properties of *F. boothii* strains and dsRNA-banding profiles. (**A**) The dsRNA-banding profiles of each *F. boothii* strain (Ep-BL13, Ep-BL14 or Ep-28) and a reference pathogenic strain. *F. graminearum* s.str. Three *F. boothii* strains all carried dsRNA fragments of approximately 3 kbp. Aside from the 3-kbp dsRNA band, Ep-BL13 harboured two additional dsRNA bands of over 10 kbp and about 2.5 kbp. (**B**,**C**) The colony morphologies of *F. boothii* strains and *F. graminearum* s.str. on PDA nutrition-rich media (**B**) and SNA nutrition-poor media (**C**) that were photographed at four days after transplanting. The three *F. boothii* strains exhibited various colony morphologies in terms of colony size, the amount of aerial mycelium and pigmentation. (**D**) Growth rate of *Fusarium* strains on PDA media. Colony sizes were measured at 3–7 days post-transplantation. (**E**) Pathogenicity test I. A wheat spikelet was inoculated with *Fusarium* strains by injection and photographed at 15 days post-inoculation. The number of spikelets exhibiting symptoms is presented. (**F**) Pathogenicity test II. Wheat photographs taken as in (**E**), but after spray inoculation. Disease indices (0–100) as per Ban and Suenaga (2000) [48] are shown.

The fungal strains Ep-BL13, Ep-BL14 and Ep-28 were isolated from wheats exhibiting FHB in Ethiopia by using *Fusarium*-selecting medium, which were found to be dsRNA-positive. Agarose gel electrophoresis of dsRNA fractions extracted from these strains showed a 3 kbp common to all three strains and additional >10 and 2.5 kbp bands only in the Ep-BL13 strain (Figure 1A), thereby suggesting infections by unknown RNA mycoviruses. These fungal strains were identified as *F. boothii* based on comparison of partial *TEF1-α* and ITS sequences (data not shown) and by phylogenetic analysis of partial *TEF1-α* sequences (Figure S1).

### 3.2. Biological Characteristics of F. boothii Strains

Colony morphology of the *F. boothii* strains on nutrient-rich (PDA) and -poor (SNA) solid media varied as shown in Figure 1B,C. The Ep-BL13 strain grew most slowly on PDA, followed by Ep-BL14 and then Ep-28 (Figure 1B,D). The strains also exhibited different phenotypes on SNA, with Ep-BL13 and Ep-N28 growing faster and slower than those on PDA, respectively (Figure 1C). The pathogenicity of the three *F. boothii* isolates and of a reference *F. graminearum* s.str. strain on wheat head was investigated with injection and spray methods. Plants inoculated with *F. boothii* Ep-BL14 and Ep-28 exhibited typical FHB symptoms similar to that of the plant infected by *F. graminearum* s.str., whereas the Ep-BL13 showed apparently weak virulence (Figure 1E,F). The effects of the dsRNA elements on host fungal growth and pathogenicity to wheats are yet to be determined.

### 3.3. Analysis of Viral Sequences: A Tymo-Like Virus

**Figure 2:**
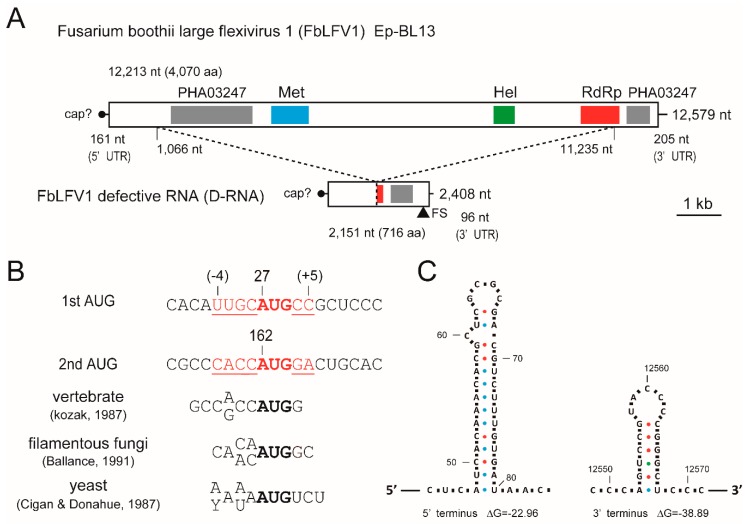
Schematic representation of the genome structure of the large *F. boothii* mycovirus. (**A**) Genome maps of FbLFV1. Thick bars are non-coding sequences and open boxes represent ORFs. Colour-highlighted boxes in ORFs represent the predicted protein domains as indicated. The length of the whole genome, ORF and UTR are given. The deleted region of FbLFV1 in defective RNA (D-RNA) is denoted by the dashed lines and nucleotide positions. Met, methyltransferase domain; Hel, helicase domain; RdRp, RNA-dependent RNA polymerase domain, FS, frame-shift. (**B**) Comparison of nucleotide contexts surrounding potential initiation codons (red bold letters, potential initiation codon; red underlined letters, surrounding nucleotides) of FbLFV1. Favourable contexts of initiation codon in vertebrate, filamentous fungi and yeast are shown as references. (**C**) Structure of terminal, non-coding RNA regions of FbLFV1. 5′- and 3′-terminal sequences of the genomes of the FbLFV1 were analysed with secondary structure prediction tools. Representatives of putative stem-loop structures were depicted. Dots in different colors indicate hydrogen bonds between different base pairs: blue, A-U pairs; red, G-C pairs; green, G-U pair.

The genome organisation of the coding strand of the largest dsRNA segment harboured in Ep-BL13 is shown in Figure 2A. The complete genomic sequence of the virus was 12,579 nt in length and the G + C content of the dsRNA is 64.93% (accession No. LC425115). A BLASTX search of the whole nucleotide sequence showed that the highest amino acid (aa) identity (35%) was to the replicase of BVF, which is the sole member of the family *Gammaflexiviridae* (Table 1). Based on this result, the virus was tentatively named Fusarium boothii large flexivirus 1 (FbLFV1). The whole genome of FbLFV1 encompasses a single large ORF putatively encoding a polypeptide of 4070 aa (162–12,374 nt) with a calculated molecular mass of 443.6 kDa. However, BVF, the closest virus to FbLFV1 in a BLAST search, possesses two ORFs encoding a putative replicase (a potential readthrough product) and a putative CP. Methyltransferase (Met), helicase (Hel) and RNA-dependent RNA polymerase (RdRp) domains of the FbLFV1 polypeptide showed low aa identities (22–36%, 20–38% and 25–37%, respectively) to those of representative viruses in the order *Tymovirales* and related mycoviruses, as shown in Table 1. Three replicase domains, Met, Hel and RdRp, are located in this order, from N to C terminus, and this is typical of the “alphavirus-like” superfamily that includes many other plant-infecting viruses (Figure 2A) [59]. There were two regions in the polypeptide with similarity to a PHA03247 domain (N-terminal part, E-value: 3.94 × e^−6^; C-terminal part, E-value: 5.01 × e^−3^), which was found in the large tegument protein UL36 of herpesviruses (a large dsDNA virus), although the function of PHA03247-like domains in FbLFV1 is unknown.

The initiation codon at 162–164 nt of the large ORF was in a favourable context, CACCAUGGa, for filamentous fungi, although another potential initiation codon at 27–29 nt appeared in frame to the ORF with a weak context uugCAUGcG (capital and small letters represent good and poor residues in the context, respectively; underlined letters are the putative initiation codon; Figure 2B) [60,61,62]. FbLFV1 had short 5′ and 3′ untranslated regions (UTRs) that are 161 and 205 nt, respectively (Figure 2A). Although experimental evidence has not been obtained, the presence of a Met domain suggested the capping of 5′-end of FbLFV1 genomic RNA. The Met domain of the members of the order *Tymovirales* is generally located at the extreme N-terminal region of the replicase but that of FbLFV1 was distant from N-terminus of the polypeptide (aa positions 1261–1528) (Figure 2A).

### 3.4. Presence of a Defective Segment of FbFV1 in the Ep-BL13

The cDNA-library-based sequencing of the approximately 2.5 kbp dsRNA revealed that the segment was of a defective RNA (D-RNA) of FbLFV1. The total length of the dsRNA was 2408 bp, and the positive-sense RNA retained an ORF encoding polypeptide with a potential molecular mass of 76.8 kDa (716 aa; Figure 2A) (accession No. LC425116). This occurred because of the loss of aa 303–3693 of the FbLFV1 large polypeptide (removal of FbLFV1 genome positions 1066–11,235 in-frame) and the addition of 36 aa because of a frame-shift from a single nucleotide deletion at −5 nt position relative to the original termination codon, leading to a new downstream stop codon. Since the RNA sequence was almost identical to the corresponding region of FbLFV1 and the junction of the upstream/downstream parts was uniform, this D-RNA of FbLFV1 might have arisen very recently, but at least before the isolation of Ep-BL13. The segment has the potential to facilitate viral replication because the coded protein retained partial RdRp motifs (122 aa residues of C-terminal end; Figure 2A); however, it is also possible that it may interfere with replication. As BVF is reported to carry similar D-RNA species with unknown function [39], it is interesting to investigate the significance of D-RNAs in life cycles of mycoviruses in the order *Tymovirales*.

### 3.5. Predicted RNA Structure of the Terminal Regions of FbLFV1 Genome

The UTRs of RNA viruses generally carry important *cis*-elements that form secondary/tertiary structures and/or distant base-pairing. In many cases, these are essential for encapsidation, transcription, translation and replication. To investigate whether FbLFV1 contains such structures, UTR sequences were subjected to secondary structure prediction using mfold software. The 5′ UTR of FbLFV1 did not consistently exhibit stem loops in several outputs, except for the one shown in Figure 2C. Similarly, those of the 3′ UTR were mostly inconsistent but a stem-loop predicted near the 3′-end was the most probable (Figure 2C). It is known that some members of *Tymoviridae*, such as turnip yellow mosaic virus (TYMV), have tRNA-like structures (TLS) in their 3′-UTR that play an important role in viral replication [63,64]. Given that TLSs are difficult to predict (e.g., the TYMV-TRL (6234–6318 nt, NC_004063) was not detected by tRNAscan-SE software [65]), more extensive base-pairing analysis of the FbLFV1 3′-end should be conducted before drawing any definitive conclusions.

### 3.6. Molecular Phylogenetic Analysis of FbLFV1

**Figure 3:**
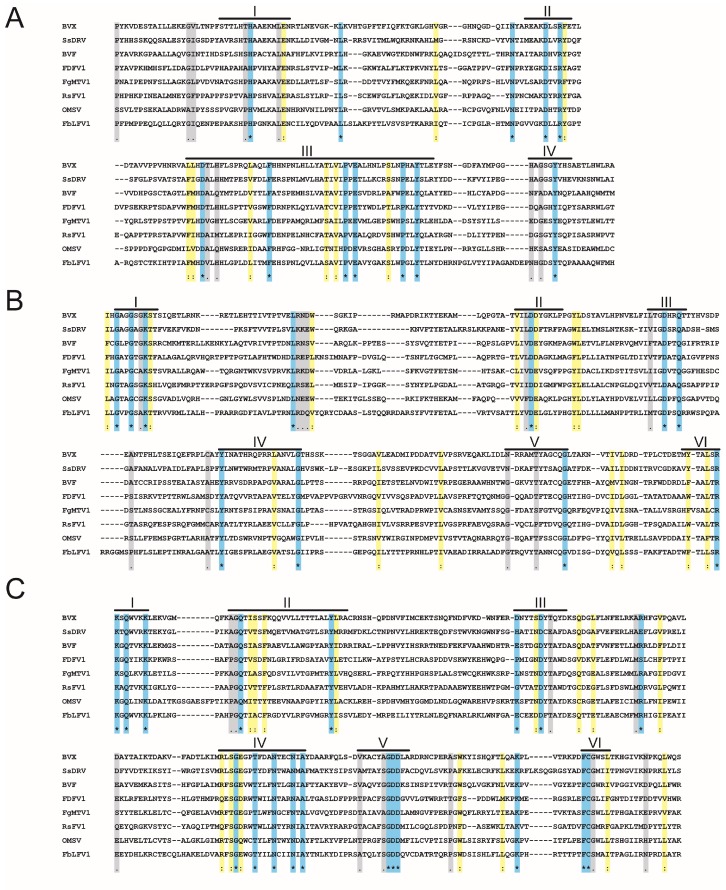
Amino acid alignment of Met, Het and RdRp domains of FbLFV1. The positions of the motifs are indicated by lines above the sequence alignments with the motif numbers from I to VI. Identical residues are colour-highlighted with blue and indicated by asterisks; conserved and semi-conserved amino acid residues are colour-highlighted with yellow and gray, and by colons and dots, respectively. (**A**–**C**) Deduced aa sequences corresponding to Met (**A**), Hel (**B**) and RdRp (**C**) domains were extracted from the FbLFV1 large polypeptide and aligned with those from mycoviruses of the order Tymovirales. Abbreviations of viral names are the same as in Table 1.

**Figure 4:**
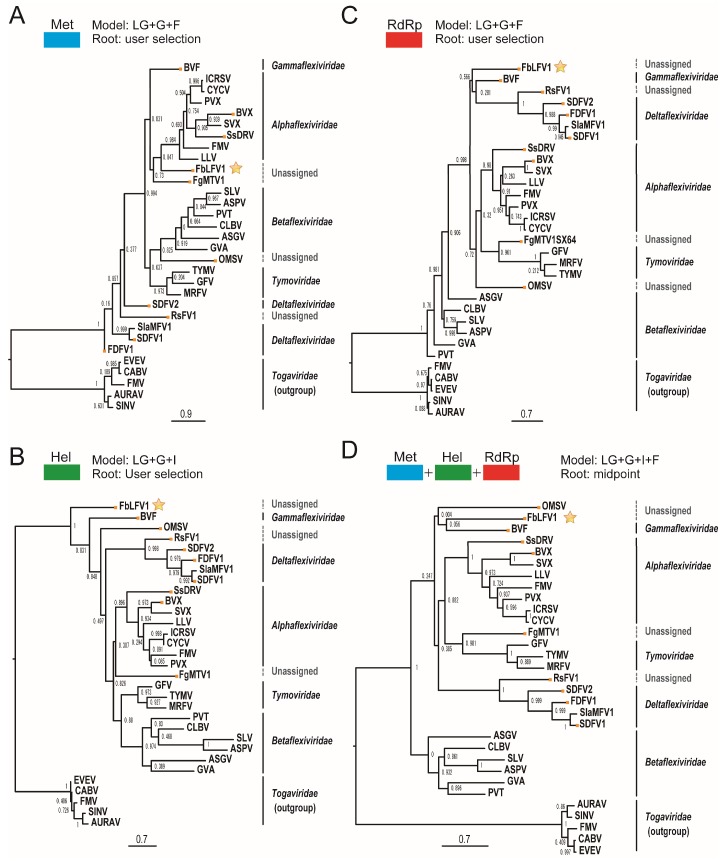
Phylogenetic analyses of FbLFV1. (**A**–**C**) Phylogenetic trees were constructed based on the alignment of aa sequences of Met (**A**), Hel (**B**) and RdRp (**C**) domains. The ML trees with the best model and rooting methods are shown. Numbers at the nodes represent branch supporting values with an aLRT SH-like method (>0.9 is significant). (**D**) Phylogenetic tree of Met + Hel + RdRp flanking sequences. Yellow dots indicate mycoviruses and stars indicate FbLFV1. The sequences used for (**A**–**C**) were joined and subjected to alignment and phylogenetic analyses as in (**A**–**C**). Abbreviations of viral names and accession numbers are listed in Table 1.

Amino acid sequences of whole FbLFV1 replicase and representative mycoviruses in the order *Tymovirales*, including taxonomically unassigned members, were aligned and the Met, Hel and RdRp domains were extracted (Table 1 and Figure 3). These three domains were found to be well conserved in the deduced protein sequence of FbLFV1 replicase. The evolutionarily conserved relationship of FbLFV1 to the *Tymovirales* members was characterised by maximum likelihood (ML) phylogenetic analyses based on the alignments of each of the three domains (Figure 4). As expected from BLAST analyses, the FbLFV1 Met Hel and RdRp domains were distantly associated with those of viruses in the order *Tymovirales*, with relatively close relationship to a gammaflexivirus (Figure 4A–C). The concatenate sequence of these domains finally revealed that FbLFV1 was placed independently from the clades of established families in the order *Tymovirales*, although the branching support value was not sufficiently high in the ML tree (Figure 4D). Based on these phylogenetic analyses, FbLFV1 is expected to belong to a novel virus species “Fusarium boothii large flexivirus 1” that may form an independent genus, or even a new virus family, in the order *Tymovirales*.

### 3.7. Analysis of Viral Sequences: Three Mitoviruses

**Figure 5:**
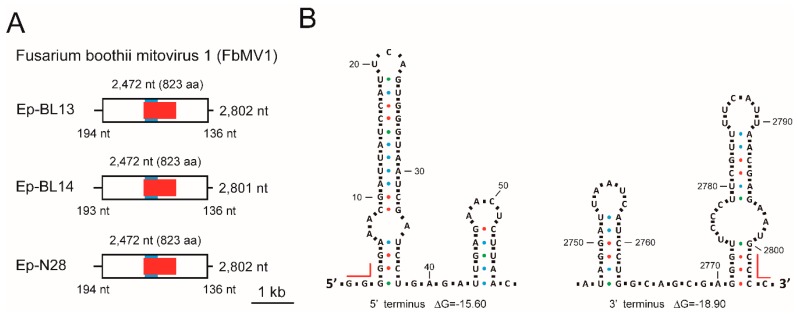
Schematic representation of the genome structure of *F. boothii* mitoviruses. (**A**) Genome maps of FbMV1. Thick bars are non-coding sequences and open boxes represent ORFs. Highlighted boxes in ORFs represent the predicted RdRp (red) and AroE (blue) domains. The length of the whole genome, ORF and UTR are given. (**B**) Structure of terminal, non-coding RNA regions of FbMV1. 5′-and 3′-terminal sequences of the genomes of FbMV1 Ep-BL13 were analysed with secondary structure prediction tools. Representatives of putative stem-loop structures were depicted. Red lines indicate nucleotide residues that potentially form base-pairing between 5′- and 3′-ends. Dots in different colours indicate hydrogen bonds between different base pairs: blue, A-U pairs; red, G-C pairs; green: G-U pairs.

The full-length sequences of the 3 kbp-dsRNAs in *F. boothii* strains Ep-BL13, Ep-BL14 and Ep-N28 were obtained by cDNA library sequencing and 3′ RLM-RACE analyses (accession Nos. LC425112, LC425113 and LC425114, respectively). The viral dsRNAs accumulated in strains Ep-BL13 and Ep-N28 were 2802 bp long and that in strain Ep-BL14 was 2801 bp long. Each positive sense strand sequence possessed a single ORF potentially encoding RdRp when using mitochondrial codon usage (Figure 5A). The length of the ORF and 3′-UTRs was the same among three sequences, being 2472 nt (823 aa) and 136 nt, respectively (Figure S2). A few nucleotide deletions at different positions in the 5′-UTRs were found in the three viral sequences; nevertheless, the 5′-UTR of Ep-BL13 and Ep-N28 were of the same length (194 nt), whereas that of strain Ep-BL14 was 1 nt shorter (193 nt; Figure S2). The nucleotide similarities between these three viruses were 98% in any combinations. A BLASTP search found that the highest aa sequence identity of the RdRp coded by three viral RNAs of Ep-BL13, Ep-BL14 and Ep-N28 were 41% (*E*-value = 1 × e^−158^), 40% (*E*-values = 8× e^−158^) and 41% (*E*-value = 2× e^−159^), respectively, to the RdRp sequence of a tentative mitovirus of uncertain origin, soybean leaf-associated mitovirus 5. The highest aa identity with a definitive mitovirus species was 30% to Ophiostoma novo-ulmi mitovirus 3a (OnuMV3a). Thus, these 3 kbp-dsRNAs are apparently replication intermediates of new mitovirus strains that are closely related to each other, and these viruses were tentatively termed Fusarium boothii mitovirus 1 (FbMV1).

Some mitoviruses form a long pan-handle structure between the 5′ and 3′ termini [66]. In the terminal regions of FbMV1 genome, tetra nucleotides at extreme termini 5′-GGGG and CCCC-3′ may have potential to form base pairs. Another common characteristic of mitovirus genomes is the presence of stem-loop structures at near the terminal regions. Both the 5′ and 3′ UTRs of the FbMV1 genome clearly folded into two potential stem-loop structures, as shown in Figure 5B. None of the sequences was predicted to form pseudoknot.

### 3.8. Molecular Phylogenetic Analysis of Mitoviral RdRps

**Figure 6:**
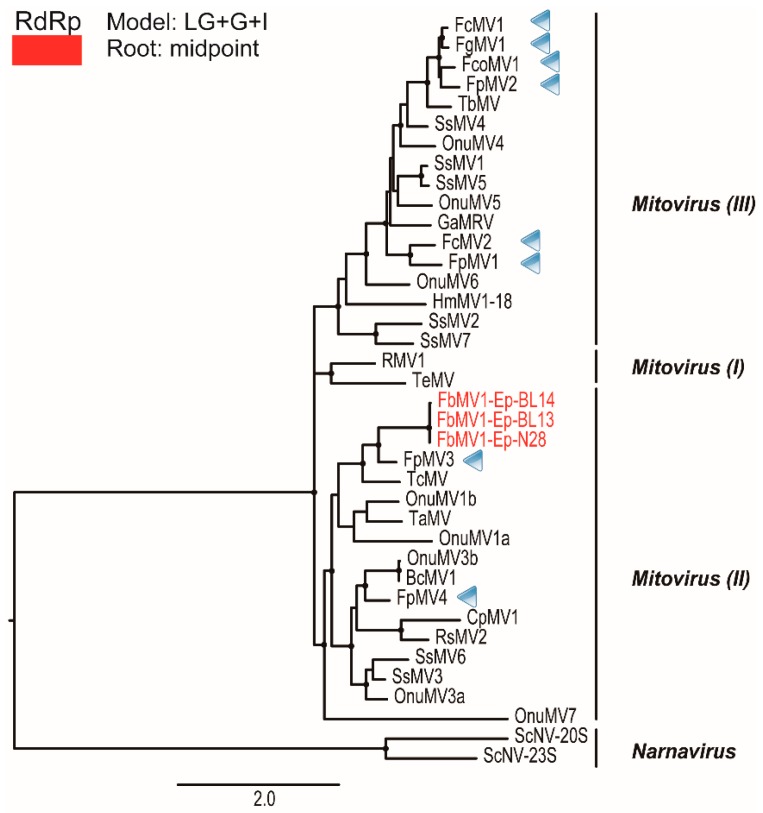
Phylogenetic analyses of FbMV1. A phylogenetic tree was constructed based on the alignment of RdRp domain sequences of narnaviruses, mitoviruses and FbMV1 strains. The midpoint-rooted ML tree is shown. Dot at the nodes represents reliable aLRT values over 0.9 for branch supporting. The best model of the ML tree constructs is indicated at the upper left. Clade clustering is referred to a previous report by Xu et al. (2015) [34]. Blue arrowheads indicate mitoviruses isolated from the *Fusarium* species. Abbreviations of viral names and accession numbers are listed in Table S1. Red letters represent mitoviruses characterized in this study.

Phylogenetic analysis of the RdRps of the three new mitoviruses and some representative mitoviruses and narnaviruses (members of the family *Narnaviridae*) was conducted using PhyML (Figure 6 and Table S1). According to Xu et al. (2015) [34], mitoviruses are divided into three major clades I, II and III, and the new mitoviruses were included in clade II accommodating OnuMV3a. Mitoviruses found from *Fusarium* species have been mostly grouped into clade III, however, FbMV1 together with recently reported Fusarium poae mitovirus 3 and 4 (FpMV3 and FpMV4 in Figure 6) [25] exemplified a broad diversity of *Fusarium* mitoviruses. Taken together, these data suggest that the three viruses characterised in this study are strains of a new virus species Fusarium boothii mitovirus 1 in the genus *Mitovirus* in the family *Narnaviridae.* This is consistent with the species demarcation criteria within the genus *Mitovirus* (more than 90% sequence identity with RdRp refers strains) in the 9th report of ICTV [29].

## 4. Discussion

Virus hunting from fungal hosts has been widely conducted, and recent advances in sequencing technologies has accelerated the discovery of novel types of mycoviruses [1,17,25,43,67]. The virus-like sequences mining from NGS-assisted metadata are highly useful in understanding the diversity and evolution of viruses although there is an argument as to what kind of information can validate the presence of “actual viruses” and their classification, even within ICTV [68]. Indeed, recent examples of virus characterization in *Rosellinia necatrix* and *S. sclerotiorum* revealed identification of novel mycoviruses including taxonomically unique individuals, and demonstrated effectiveness of NGS [69,70]. Hence, the accumulation of more virome data from African biological materials will facilitate our understanding of mycovirus diversity and may lead to discovery of unexpectedly unique populations of mycoviruses, as is the case for FbLFV1. There are many barriers in the establishment of effective virocontrol of herbaceous annual plant diseases, including FHB, as discussed by Pearson et al. [71]. Thus, it will be important to screen for virocontrol agents which have much better properties such as higher transmissibility and broader cross-species specificity.

**Figure 7:**
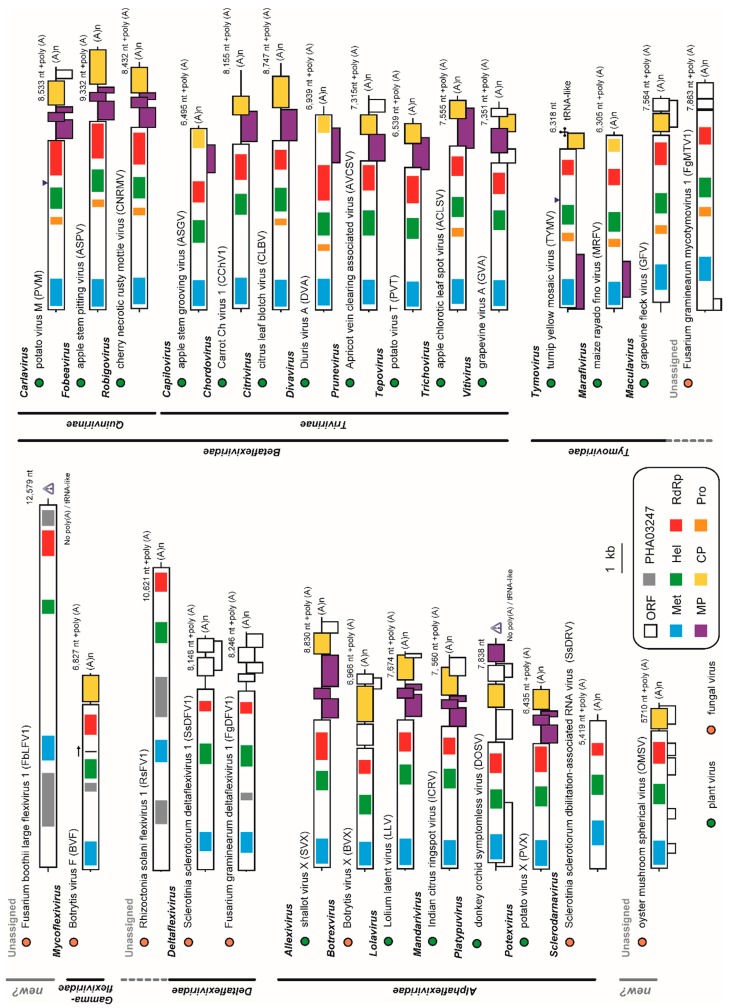
Comparison of genomic structures of the *Tymovirales* members and related unclassified viruses. Representative members of *Tymovirales* were selected as shown in Table 1 and their genomic structures are illustrated. Viral families, subfamilies and genera are shown in bold letters, and ‘new?’ represents a potential new taxon. Colour differentiated symbols are as indicated. The arrow on BVF replicase indicates potential readthrough translation. The blue triangle points to a known proteolytic cleavage site. Gray triangles at 3′-end of FbLFV1 and DOSV genomes indicate uncommon form that is not polyadenylated or tRNA-like structured.

Characterization of mycoviruses has significantly contributed to the taxonomy of viruses including the order *Tymovirales*. Those include creation of the genus *Mycoflexivirus* and the family *Gammaflexiviridae* (BVF); creation of the genus *Deltaflexivirus* and the family *Deltaflexiviridae* (SsDFV1, SsDFV2, FgDFV1 and soybean leaf-associated mycoflexivirus 1); creation of the genera *Sclerodarnavirus* and *Botrexvirus* under the family *Alphaflexiviridae* (SsDARV and BVX, respectively); and proposal of new genus Mycotymovirus under the family *Tymoviridae* (FgMTV1) (Table 1; Figure 7) [20,27,39,40,41,42,67]. This study additionally reports a novel member of the order *Tymovirales* that has a potential to further expand taxonomic diversity in the order.

FbLFV1 showed similar characteristic to and distinct features from present five families in the order as follows. The careful assessment of phylogenetic relationship between FbLFV1 and other *Tymovirales* members revealed its close-yet-distinct correlations to the gammaflexivirus BVF and deltaflexiviruses (Figure 3 and Figure 4). The presence of PHA03247 domain in these viruses further supported their evolutionally close association. However, the genome length and organization of FbLFV1 and BVF differed from each other (FbLFV1, 12,579 nt with single ORF vs. BVF, 6827 nt + poly-A tail with two ORFs) [39]. The absence of CP or CP domain of trichovirus/tymoviruses (see those of apple stem grooving virus, Diuris virus A and maize rayado fino marafivirus in Figure 7) in the FbLFV1 large polypeptide is highly possible and this feature is similar to deltaflexiviruses, an exceptional alphaflexivirus SsDRV and a tymo-like virus FgMTV1, highlighting the uniqueness of capsid-less nature of some mycoviruses, which is often observed (Figure 7) [20,27,38,41,42,45,67]. All *Tymovirales* viruses possess poly-A tail at the 3′ end with exceptions of those of the genus *Tymovirus* in the family *Tymoviridae,* which have TLSs, and of the genus *Platypuvirus* in the family *Alphaflexiviridae*, which have no obvious RNA structures (Figure 7) [72]. The 3′ end of FbLFV1 genome does has neither a poly-A tail nor TLS, further discriminating FbLFV1 from established genera and families in the order *Tymovirales* (Figure 2 and Figure 7). In contrast, 5′ ends of *Tymovirales* virus genomes poorly conserve characteristics, except for the cap structure, across the families. In the genus *Tymovirus*, 5′ UTRs generally carry one to four protonatable hairpins nearby initiation codons that are probably required for dicistronic expression of overlapping ORFs (Figure 7, *Tymovirus*) [73]. The stem-loop structure predicted in 5′ UTR of FbLFV1 was placed at 81 nt upstream of start codon (Figure 2C) and there is no potential overlapping ORF, suggesting a distinct role of the structure in multiplication process of FbLFV1.

The genome length of the members in the order *Tymovirales* ranges from about 5.9 to 9.0 kb [59] whereas the recently reported unassigned member, a *Rhizoctonia* virus RsFV1, has the largest genome (10,621 nt + poly-A tail) [38]. The discovery of FbFLV1 increases the maximum genome size in the order by about 2 kb. RsFV1 and FbFLV1 share a similar genomic structure: the large RNA genome of over 10 kb; the single large ORF encoding replicase; the presence of a functionally unknown region at N-terminus in the replicase; and a potential of capsid-less nature (Figure 7). The N-terminal regions contain a PHA03247 protein motif that is similar to a portion of the UL36 large tegument protein found in herpesviruses that has also been found from other mycoviruses BVF and SsDRV (Figure 7) [74]. Despite the above-mentioned similarity, RsFV1 is evolutionally closer to *deltaflexivirus*, whereas FbLFV1 is phylogenetically independent. In addition, a taxonomically floating virus, OMSV, appeared to be monophyletic, despite its apparently tymovirus (*Tymoviridae*)-like characteristics with respect to genome organisation and spherical virion structure (Figure 4 and Figure 7) [44]. Thus, FbLFV1 and OMSV will become key viruses when taxonomic reconsideration within the order *Tymovirales* is required.

The strains of FbMV1 were found in three independent *F. boothii* isolates, and TcMV exhibited a high similarity to FbMV1 (35%). It has been reported that the TcMV RdRp has an AroE domain, which is one of the domains on AROM protein encoded by *Saccharomyces cerevisiae* [75]. AROM is a pentafunctional polypeptide that catalyses a sequence of five reactions in prechorismate polyaromatic amino acid biosynthesis, which is the former half of shikimate pathway. Although the function of the AroE-like domain in TcMV RdRp is unclear, similar sequences have been found in phylogenetically close relatives such as CpMV1 and OnuMV3a, as well as FbMV1 (Figure 5 and Figure S3), further supporting the involvement of FbMV1 in the mitovirus group of clade II. TcMV was identified in a basidiomycete fungus, *Thanatephorus cucumeris* (anamorph *Rhizoctonia solani*), which is phylogenetically very distant from ascomycetous *Fusarium* species. These points support an earlier hypothesis by Deng et al. [76] that mitoviruses have evolved not only through co-evolution with the viruses strictly confined within their host fungi and close relatives, but also through host-switching and adaptation to phylogenetically distant hosts via unknown routes of transmissions.

Overall, this study sequenced three isolates of a new mitovirus FbMV1 harboured in three independent *F. boothii* strains, as well as a novel tymo-like virus, FbLFV1, with the largest genome size thus far described in the order *Tymovirales*. Detailed analyses of gene expression, potential polyprotein processing, particle formation and pathogenicity of FbLFV1 are the next challenges to understand the properties of this new taxon in the order *Tymovirales*. Biological property of FbLFV1 may be able to be assessed by protoplast-fusion method as reported for FgV1-DK21 [77].

## Figures and Tables

**Table 1:** List of viruses in the order Tymovirales and related unassigned viruses with similarity to FbLFV1.

Taxon	Virus Name (Abbreviation)	Accession	Met *	Hel *	RdRp *
*Alphaflexiviridae*					
*Allexivirus*	Shallot virus X (SVX)	NC_003795	58/198 (29%)	37/154 (24%)	74/221 (33%)
*Botrexvirus*	Botrytis virus X (BVX)	NC_005132	60/196 (30%)	45/178 (25%)	76/221 (34%)
*Lolavirus*	Lolium latent virus (LLV)	NC_010434	64/193 (33%)	50/241 (20%)	80/238 (33%)
*Mandarivirus*	Indian citrus ringspot virus (ICRV)	NC_003093	59/162 (36%)	63/244 (25%)	84/229 (36%)
*Platypuvirus*	Donkey orchid symptomless virus (DOSV)	NC_022894	60/201 (29%)	49/189 (25%)	69/225 (30%)
*Potexvirus*	Potato virus X (PVX)	NC_011620	60/198 (30%)	58/245 (23%)	79/227 (34%)
*Sclerodarnavirus*	Sclerotinia sclerotiorum debilitation-associatedRNA virus (SsDARV)	NC_007415	63/198 (31%)	49/191 (25%)	71/227 (31%)
*Betaflexiviridae*					
*Quinvirinae*					
*Carlavirus*	Potato virus M (PVM)	NC_001361	55/217 (25%)	35/127 (27%)	62/224 (27%)
*Foveavirus*	Apple stem pitting virus (ASPV)	NC_001749	60/222 (27%)	17/45 (37%)	63/222 (28%)
*Robigovirus*	Cherry necrotic rusty mottle virus (CNRMV)	NC_002468	52/224 (23%)	44/159 (27%)	61/219 (27%)
*Trivirinae*					
*Capillovirus*	Apple stem grooving virus (ASGV)	NC_003462	64/214 (29%)	33/97 (34%)	72/231 (31%)
*Chordovirus*	Carrot Ch virus 1 (CChV)	NC_025469	54/214 (25%)	33/118 (27%)	66/225 (29%)
*Citrivirus*	Citrus leaf blotch virus (CLBV)	NC_003877	46/205 (22%)	46/160 (28%)	68/223 (30%)
*Divavirus*	Diuris virus A (DVA)	NC_019029	53/217 (24%)	26/90 (28%)	66/224 (29%)
*Prunevirus*	Apricot vein clearing associated virus (AVCSV)	NC_023295	49/183 (26%)	24/62 (38%)	66/224 (29%)
*Tepovirus*	Potato virus T (PVT)	NC_011062	56/186 (30%)	34/115 (29%)	67/219 (30%)
*Trichovirus*	Apple chlorotic leaf spot virus (ACLSV)	NC_001409	62/195 (31%)	43/172 (25%)	64/218 (29%)
*Vitivirus*	Grapevine virus A (GVA)	NC_003604	50/201 (24%)	22/57 (38%)	65/218 (29%)
*Gammaflexiviridae*					
*Mycoflexivirus*	Botrytis virus F (BVF)	NC_002604	63/193 (32%)	71/252 (28%)	82/224 (36%)
*Deltaflexiviridae*					
*Deltaflexivirus*	Sclerotinia sclerotiorum deltaflexivirus 1 (SsDFV1)	NC_038977	54/202 (26%)	61/253 (24%)	45/174 (25%)
	Sclerotinia sclerotiorum deltaflexivirus 2 (SsDFV2)	MH299810	63/222 (28%)	44/184 (23%)	59/207 (28%)
	Fusarium graminearum deltaflexivirus 1 (FgDFV1)	NC_030654	67/204 (32%)	37/157 (23%)	57/211 (27%)
	Soybean leaf-associated deltaflexivirus 1 (SlaDFV1)	KT598226	54/203 (26%)	63/260 (24%)	46/174 (26%)
*Tymoviridae*					
*Maculavirus*	Grapevine fleck virus (GFV)	NC_003347	57/203 (28%)	43/144 (29%)	54/143 (37%)
*Marafivirus*	Maize rayado fino virus (MRFV)	NC_002786	58/197 (29%)	57/201 (28%)	45/140 (32%)
*Tymovirus*	Turnip yellow mosaic virus (TUMV)	NC_004063	56/196 (28%)	58/214 (27%)	61/204 (29%)
Unassigned					
Mycotymovirus	Fusarium graminearum mycotymovirus 1 (FgMTV1)	KT360947	60/196 (30%)	57/197 (26%)	73/219 (33%)
Unassigned	Rhizoctonia solani flexivirus 1 (RnFV1)	NC_030655	57/198 (28%)	53/228 (23%)	65/224 (29%)
Unassigned	Rhizoctonia solani flexivirus 2 (RnFV1)	KX349069	N/A **	N/A **	50/177 (28%)
Unassigned	Oyster muschroom spherical virus (OMSV)	AY182001	48/189 (25%)	61/237 (25%)	73/219 (33%)
Unassigned	Fusarium boothii large flexivirus 1 (FbLFV1)	LC425115			

* Results of homology search against FbLFV1 for amino acid cover region and identity are shown. ** N/A indicates “not applicable” due to the unavailability of homologous sequences.

## Supplementary Materials

The following are available online at http://www.mdpi.com/1999-4915/10/11/584/s1, Table S1. List of viruses in the family *Narnaviridae* and related unassigned members used in the phylogenetic study shown in Figure 6. Figure S1. Phylogenetic analysis of partial *TEF1-α* gene sequences derived from *Fusarium* samples. Products from the genomic PCR targeting *TEF1-α* were subjected to direct sequencing and the resultant nucleotide sequences were aligned in MAFFT ver. 7 with publicly available sequences of various *F. graminearum* species complex members. The alignment was used for maximum likelihood phylogenetic analysis with the recommended model shown at upper left. Figure S2. Nucleotide sequence alignment of three FbMV1 strains Ep-BL13, Ep-BL14 and Ep-N28. The entire nucleotide sequences of FbMV1 strains aligned with MAFFT ver.7. Small letters, sequence of non-coding region; capital letters, replicase coding region. Figure S3. The putative AROM domains in mitovirus RdRps. The amino acid sequence of AroE domain of AROM protein encoded by *S. cerevisiae*, and corresponding sequences of FbMV1, TcMV and close relatives were aligned with MAFFT ver.7.

## References

[1] Ghabrial S.A., Castón J.R., Jiang D., Nibert M.L., Suzuki N. (2015). 50-plus years of fungal viruses. Virology.

[2] Pan J., Dong L., Lin L., Ochoa W.F., Sinkovits R.S., Havens W.M., Nibert M.L., Baker T.S., Ghabrial S.A., Tao Y.J. (2009). Atomic structure reveals the unique capsid organization of a dsRNA virus. Proc. Natl. Acad. Sci. USA.

[3] Hillman B.I., Cai G. (2013). The Family Narnaviridae: Simplest of RNA Viruses. Adv. Virus Res..

[4] Hillman B.I., Suzuki N. (2004). Viruses of the chestnut blight fungi, *Cryphonectria parasitica*. Adv. Virus Res..

[5] Suzaki K., Ikeda K., Sasaki A., Kanematsu S., Matsumoto N., Yoshida K. (2005). Horizontal transmission and host-virulence attenuation of totivirus in violet root rot fungus *Helicobasidium mompa*. J. Gen. Plant Pathol..

[6] Moleleki N., van Heerden S.W., Wingfield M.J., Wingfield B.D., Preisig O. (2003). Transfection of *Diaporthe perjuncta* with Diaporthe RNA virus. Appl. Environ. Microbiol..

[7] Wu M.D., Zhang L., Li G.Q., Jiang D.H., Hou M.S., Huang H.C. (2007). Hypovirulence and Double-Stranded RNA in *Botrytis cinerea*. Phytopathology.

[8] Castro M., Kramer K., Valdivia L., Ortiz S., Castillo A. (2003). A double-stranded RNA mycovirus confers hypovirulence-associated traits to *Botrytis cinerea*. FEMS Microbiol. Lett..

[9] Chu Y.M., Jeon J.J., Yea S.J., Kim Y.H., Yun S.H., Lee Y.W., Kim K.H. (2002). Double-stranded RNA mycovirus from *Fusarium graminearum*. Appl. Environ. Microbiol..

[10] Hong Y., Dover S.L., Cole T.E., Brasier C.M., Buck K.W. (1999). Multiple Mitochondrial Viruses in an Isolate of the Dutch Elm Disease Fungus *Ophiostoma novo-ulmi*. Virology.

[11] Yu X., Li B., Fu Y., Jiang D., Ghabrial S.A., Li G., Peng Y., Xie J., Cheng J., Huang J. (2010). A geminivirus-related DNA mycovirus that confers hypovirulence to a plant pathogenic fungus. Proc. Natl. Acad. Sci. USA.

[12] De Wet J., Bihon W., Preisig O., Wingfield B.D., Wingfield M.J. (2011). Characterization of a novel dsRNA element in the pine endophytic fungus *Diplodia scrobiculata*. Arch. Virol..

[13] Preisig O., Wingfield B.D., Wingfield M.J. (1998). Coinfection of a Fungal Pathogen by Two Distinct Double-Stranded RNA Viruses. Virology.

[14] Vainio E.J., Martínez-Álvarez P., Bezos D., Hantula J., Diez J.J. (2015). *Fusarium circinatum* isolates from northern Spain are commonly infected by three distinct mitoviruses. Arch. Virol..

[15] Smit W.A., Wingfield B.D., Wingfield M.J. (1996). Reduction of laccase activity and other hypovirulence-associated traits in dsRNA-containig strains of *Diaporthe ambigua*. Phytopathology.

[16] Preisig O., Moleleki N., Smit W.A., Wingfield B.D., Wingfield M.J. (2000). A novel RNA mycovirus in a hypovirulent isolate of the plant pathogen *Diaporthe ambigua*. J. Gen. Virol..

[17] Coetzee B., Freeborough M.J., Maree H.J., Celton J.M., Rees D.J.G., Burger J.T. (2010). Deep sequencing analysis of viruses infecting grapevines: Virome of a vineyard. Virology.

[18] Darissa O., Adam G., Schäfer W. (2012). A dsRNA mycovirus causes hypovirulence of *Fusarium graminearum* to wheat and maize. Eur. J. Plant Pathol..

[19] Wang L., He H., Wang S., Chen X., Qiu D., Kondo H., Guo L. (2018). Evidence for a novel negative-stranded RNA mycovirus isolated from the plant pathogenic fungus *Fusarium graminearum*. Virology.

[20] Li P., Lin Y., Zhang H., Wang S., Qiu D., Guo L. (2016). Molecular characterization of a novel mycovirus of the family Tymoviridae isolated from the plant pathogenic fungus *Fusarium graminearum*. Virology.

[21] Li P., Zhang H., Chen X., Qiu D., Guo L. (2015). Molecular characterization of a novel hypovirus from the plant pathogenic fungus *Fusarium graminearum*. Virology.

[22] Yu J.S., Lee K.M., Son M.I., Kim K.H. (2011). Molecular Characterization of Fusarium Graminearum Virus 2 Isolated from *Fusarium graminearum* Strain 98-8-60. Plant Pathol. J..

[23] Kwon S.J., Lim W.S., Park S.H., Park M.R., Kim K.H. (2007). Molecular Characterization of a dsRNA Mycovirus, *Fusarium graminearum* Virus-DK21, which Is Phylogenetically Related to Hypoviruses but Has a Genome Organization and Gene Expression Strategy Resembling Those of Plant Potex-like Viruses. Mol. Cells.

[24] Yu J.S., Kwon S.J., Lee K.M., Son M., Kim K.H. (2009). Complete nucleotide sequence of double-stranded RNA viruses from *Fusarium graminearum* strain DK3. Arch. Virol..

[25] Osaki H., Sasaki A., Nomiyama K., Tomioka K. (2016). Multiple virus infection in a single strain of *Fusarium poae* shown by deep sequencing. Virus Genes.

[26] Wang S., Kondo H., Liu L., Guo L., Qiu D. (2013). A novel virus in the family Hypoviridae from the plant pathogenic fungus *Fusarium graminearum*. Virus Res..

[27] Chen X., He H., Yang X., Zeng H., Qiu D., Guo L. (2016). The complete genome sequence of a novel Fusarium graminearum RNA virus in a new proposed family within the order Tymovirales. Arch. Virol..

[28] Marvelli R.A., Hobbs H.A., Li S., McCoppin N.K., Domier L.L., Hartman G.L., Eastburn D.M. (2014). Identification of novel double-stranded RNA mycoviruses of *Fusarium virguliforme* and evidence of their effects on virulence. Arch. Virol..

[29] King A.M.Q., Adams M.J., Carstens E.B., Lefkowitz E.J. (2011). Narnaviridae. Virus Taxonomy: Ninth Report of the International Committee on Taxonomy of Viruses.

[30] Polashock J.J., Hillman B.I. (1994). A small mitochondrial double-stranded (ds) RNA element associated with a hypovirulent strain of the chestnut blight fungus and ancestrally related to yeast cytoplasmic T and W dsRNAs. Proc. Natl. Acad. Sci. USA.

[31] Khalifa M.E., Pearson M.N. (2013). Molecular characterization of three mitoviruses co-infecting a hypovirulent isolate of *Sclerotinia sclerotiorum* fungus. Virology.

[32] Wu M., Zhang L., Li G., Jiang D., Ghabrial S.A. (2010). Genome characterization of a debilitation-associated mitovirus infecting the phytopathogenic fungus *Botrytis cinerea*. Virology.

[33] Xie J., Ghabrial S.A. (2012). Molecular characterizations of two mitoviruses co-infecting a hyovirulent isolate of the plant pathogenic fungus *Sclerotinia sclerotiorum*. Virology.

[34] Xu Z., Wu S., Liu L., Cheng J., Fu Y., Jiang D., Xie J. (2015). A mitovirus related to plant mitochondrial gene confers hypovirulence on the phytopathogenic fungus *Sclerotinia sclerotiorum*. Virus Res..

[35] Jian J., Lakshman D.K., Tavantzis S.M. (1997). Association of Distinct Double-Stranded RNAs with Enhanced or Diminished Virulence in *Rhizoctonia solani* Infecting Potato. Mol. Plant-Microbe Interact..

[36] Martınetz P., Vainio E.J., Botella L., Hantula J., Diez J.J. (2014). Three mitovirus strains infecting a single isolate of *Fusarium circinatum* are the first putative members of the family *Narnaviridae* detected in a fungus of the genus *Fusarium*. Arch. Virol..

[37] Osaki H., Sasaki A., Nomiyama K., Sekiguchi H., Tomioka K., Takehara T. (2015). Isolation and characterization of two mitoviruses and a putative alphapartitivirus from *Fusarium* spp.. Virus Genes.

[38] Bartholomäus A., Wibberg D., Winkler A., Pühler A., Schlüter A., Varrelmann M. (2017). Identification of a novel mycovirus isolated from *Rhizoctonia solani* (AG 2-2 IV) provides further information about genome plasticity within the order *Tymovirales*. Arch. Virol..

[39] Howitt R.L.J., Beever R.E., Forster R.L.S., Pearson M.N. (2001). Genome characterization of Botrytis virus F, a flexuous rod-shaped mycovirus resembling plant ‘potex-like’ viruses. J. Gen. Virol..

[40] Howitt R.L.J., Beever R.E., Pearson M.N., Forster R.L.S. (2006). Genome characterization of a flexuous rod-shaped mycovirus, Botrytis virus X, reveals high amino acid identity to genes from plant ‘potex-like’ viruses. Arch. Virol..

[41] Xie J., Wei D., Jiang D., Fu Y., Li G., Ghabrial S., Peng Y. (2006). Characterization of debilitation-associated mycovirus infecting the plant-pathogenic fungus *Sclerotinia sclerotiorum*. J. Gen. Virol..

[42] Li K., Zheng D., Cheng J., Chen T., Fu Y., Jiang D., Xie J. (2015). Characterization of a novel *Sclerotinia sclerotiorum* RNA virus as the prototype of a new proposed family within the order *Tymovirales*. Virus Res..

[43] Bartholomäus A., Wibberg D., Winkler A., Pühler A., Schlüter A., Varrelmann M. (2016). Deep sequencing analysis reveals the mycoviral diversity of the virome of an avirulent isolate of *Rhizoctonia solani* AG-2-2 IV. PLoS ONE.

[44] Yu H.J., Lim D., Lee H.S. (2003). Characterization of a novel single-stranded RNA mycovirus in *pleurotus ostreatus*. Virology.

[45] Hamid M., Xie J., Wu S., Maria S., Zheng D., Assane Hamidou A., Wang Q., Cheng J., Fu Y., Jiang D. (2018). A Novel Deltaflexivirus that Infects the Plant Fungal Pathogen, *Sclerotinia sclerotiorum*, Can Be Transmitted Among Host Vegetative Incompatible Strains. Viruses.

[46] Mackintosh C.A., Garvin D.F., Radmer L.E., Heinen S.J., Muehlbauer G.J. (2006). A model wheat cultivar for transformation to improve resistance to Fusarium Head Blight. Plant Cell Rep..

[47] Suga H., Kageyama K., Shimizu M., Hyakumachi M. (2016). A Natural Mutation Involving both Pathogenicity and Perithecium Formation in the *Fusarium graminearum* Species Complex. G3 (Bethesda)..

[48] Ban T., Suenaga K. (2000). Genetic analysis of resistance to Fusarium head blight caused by *Fusarium graminearum* in Chinese wheat cultivar Sumai 3 and the Japanese cultivar Saikai 165. Euphytica.

[49] O’Donnell K., Kistler H.C., Cigelnik E., Ploetz R.C. (1998). Multiple evolutionary origins of the fungus causing Panama disease of banana: Concordant evidence from nuclear and mitochondrial gene genealogies. Proc. Natl. Acad. Sci. USA.

[50] O’Donnell K., Cigelnik E., Nirenberg H.I. (1998). Molecular Systematics and Phylogeography of the *Gibberella fujikuroi* Species Complex. Mycologia.

[51] Andrews S. FastQC: A Quality Control Tool for High Throughput Sequence Data. https://www.bioinformatics.babraham.ac.uk/projects/fastqc/.

[52] Joshi N., Fass J. Sickle: A Sliding-Window, Adaptive, Quality-Based Trimming Tool for FastQ Files (Version 1.33). https://github.com/najoshi/sickle.

[53] Bankevich A., Nurk S., Antipov D., Gurevich A.A., Dvorkin M., Kulikov A.S., Lesin V.M., Nikolenko S.I., Pham S., Prjibelski A.D. (2012). SPAdes: A New Genome Assembly Algorithm and Its Applications to Single-Cell Sequencing. J. Comput. Biol..

[54] Finn R.D., Attwood T.K., Babbitt P.C., Bateman A., Bork P., Bridge A.J., Chang H.Y., Dosztányi Z., El-Gebali S., Fraser M. (2017). InterPro in 2017—Beyond protein family and domain annotations. Nucleic Acids Res..

[55] Zuker M. (2003). Mfold web server for nucleic acid folding and hybridization prediction. Nucleic Acids Res..

[56] Edgar R.C. (2004). MUSCLE: Multiple sequence alignment with high accuracy and high throughput. Nucleic Acids Res..

[57] Kumar S., Stecher G., Li M., Knyaz C., Tamura K., Battistuzzi F.U. (2018). MEGA X: Molecular Evolutionary Genetics Analysis across Computing Platforms. Mol. Biol. Evol..

[58] Guindon S., Dufayard J.F., Lefort V., Anisimova M., Hordijk W., Gascuel O. (2010). New Algorithms and Methods to Estimate Maximum-Likelihood Phylogenies: Assessing the Performance of PhyML 3.0. Syst. Biol..

[59] King A.M.Q., Adams M.J., Carstens E.B., Lefkowitz E.J. (2011). Tymovirales. Virus Taxonomy: Ninth Report of the International Committee on Taxonomy of Viruses.

[60] Ballance D.J. (1986). Sequences important for gene expression in filamentous fungi. Yeast.

[61] Kozak M. (1987). At least six nucleotides preceding the AUG initiator codon enhance translation in mammalian cells. J. Mol. Biol..

[62] Mark Cigan A., Donahue T.F. (1987). Sequence and structural features associated with translational initiator regions in yeast—A review. Gene.

[63] Dreher T.W. (2009). Role of tRNA-like structures in controlling plant virus replication. Virus Res..

[64] Matsuda D., Dreher T.W. (2004). The tRNA-like structure of Turnip yellow mosaic virus RNA is a 3′-translational enhancer. Virology.

[65] Lowe T.M., Chan P.P. (2016). tRNAscan-SE On-line: Integrating search and context for analysis of transfer RNA genes. Nucleic Acids Res..

[66] Osaki H., Nakamura H., Nomura K., Matsumoto N., Yoshida K. (2005). Nucleotide sequence of a mitochondrial RNA virus from the plant pathogenic fungus, *Helicobasidium mompa* Tanaka. Virus Res..

[67] Marzano S.Y.L., Domier L.L. (2016). Novel mycoviruses discovered from metatranscriptomics survey of soybean phyllosphere phytobiomes. Virus Res..

[68] Khalifa M.E., Varsani A., Ganley A.R.D., Pearson M.N. (2016). Comparison of Illumina de novo assembled and Sanger sequenced viral genomes: A case study for RNA viruses recovered from the plant pathogenic fungus *Sclerotinia sclerotiorum*. Virus Res..

[69] Mu F., Xie J., Cheng S., You M.P., Barbetti M.J., Jia J., Wang Q., Cheng J., Fu Y.P., Chen T. (2018). Virome Characterization of a Collection of *S. sclerotiorum* from Australia. Front. Microbiol..

[70] Arjona-Lopez J.M., Telengech P., Jamal A., Hisano S., Kondo H., Yelin M.D., Arjona-Girona I., Kanematsu S., Lopez-Herrera C.J., Suzuki N. (2018). Novel, diverse RNA viruses from Mediterranean isolates of the phytopathogenic fungus, *Rosellinia necatrix*: Insights into evolutionary biology of fungal viruses. Environ. Microbiol..

[71] Pearson M.N., Beever R.E., Boine B., Arthur K. (2009). Mycoviruses of filamentous fungi and their relevance to plant pathology. Mol. Plant Pathol..

[72] Wylie S.J., Li H., Jones M.G.K. (2013). Donkey Orchid Symptomless Virus: A Viral ‘Platypus’ from Australian Terrestrial Orchids. PLoS ONE.

[73] Tzanetakis I.E., Tsai C.H., Martin R.R., Dreher T.W. (2009). A tymovirus with an atypical 3′-UTR illuminates the possibilities for 3′-UTR evolution. Virology.

[74] Fan W.H., Roberts A.P.E., McElwee M., Bhella D., Rixon F.J., Lauder R. (2015). The large tegument protein pUL36 is essential for formation of the capsid vertex-specific component at the capsid-tegument interface of herpes simplex virus 1. J. Virol..

[75] Lakshman D.K., Jian J., Tavantzis S.M. (1998). A double-stranded RNA element from a hypovirulent strain of *Rhizoctonia solani* occurs in DNA form and is genetically related to the pentafunctional AROM protein of the shikimate pathway (RNA-dependent RNA polymerase). Microbiology.

[76] Deng F., Xu R., Boland G.J. (2003). Hypovirulence-Associated Double-Stranded RNA from *Sclerotinia homoeocarpa* Is Conspecific with *Ophiostoma novo-ulmi* Mitovirus 3a-Ld. Phytopathology.

[77] Lee K.M., Yu J., Son M., Lee Y.W., Kim K.H., Arkowitz R.A. (2011). Transmission of *Fusarium boothii* Mycovirus via Protoplast Fusion Causes Hypovirulence in Other Phytopathogenic Fungi. PLoS ONE.

