# Supplementary material for: Novel Mitoviruses and a Unique Tymo-Like Virus in Hypovirulent and Virulent Strains of the Fusarium Head Blight Fungus, Fusarium boothii
Source: Viruses. 2018 Oct 26;10(11):584. doi: 10.3390/v10110584 (PMC6266667; doi:10.3390/v10110584)
Supplement: Supplementary file 1 [file viruses-10-00584-s001.zip › sup materials/Mizutani_Fig-S2.pdf]

CLUSTAL format alignment by MAFFT (v7.408)

```
FbMV1_Ep-BL13   gggggaaacgattatccattcagtgggtaatcgatcctgagattgagaactcttaaccct
FbMV1_Ep-N28    -ggggaaacgattatccattcagtgggtaatcgatcctgagattgagaactcttaaccct
FbMV1_Ep-BL14   -ggggaaacgattgtccatttcgtggataatcgatcctgagattgagaactcttaaccct
                 ************.******. ****.*********************************

FbMV1_Ep-BL13   tatctcggtggattttatcttaaactcatcccaactgagggagaagtctggataactcac
FbMV1_Ep-N28    tatctcggtggattttatcttaaactcatcccaactgagggagaagtctggataactcac
FbMV1_Ep-BL14   tatctcggtggattttatcttaaactcatcccaactgagggagaagtctggataactcac
                ************************************************************

FbMV1_Ep-BL13   caaccaaagatccgaaaaactaggtccaag-tttaattacttgaattaccagttcttacg
FbMV1_Ep-N28    caaccaaagatccgaaaaactaggtccaagtttaattacttgaattaccagttcttacg
FbMV1_Ep-BL14   caaccaaagatccgaaaaactaggtccaag-tttaattacttgaattaccagttcttacg
                ****************************** *****************************

FbMV1_Ep-BL13   ctagcaccaccgcggATGAACCAGGATGGGGATCGGATCCCAAGCCTGCCCATATTACGG
FbMV1_Ep-N28    ctagcaccaccgcggATGAACCAGGATGGGGATCGGATCCCAAGCCTGCCCATATTACGG
FbMV1_Ep-BL14   ctagcaccaccgcggATGAACCAGGATGGGGATCGGATCCCAAGCCTGCCCATATTACGG
                ************************************************************

FbMV1_Ep-BL13   ACTTTAGTGCTATGTTGTTTAATCACCTTAATTATGAAATCTACTTCATTAACTCTTTTG
FbMV1_Ep-N28    ACTTTAGTGCTATGTTGTTTAATCACCTTAATTATGAAATCTACTTCATTAACTCTTTTG
FbMV1_Ep-BL14   ACTTTAGTGCTATGTTGTTTAATCACCTTAATTATGAAATCTACTTCATTAACTCTTTTG
                ************************************************************

FbMV1_Ep-BL13   AATTCAATCAAATTGTTAAAGAGTAGATCCGTAAGAAAGATGAAGATGTTTCCTTTGAGT
FbMV1_Ep-N28    AATTCAATCAAATTGTTAAAGAGTAGATCCGTAAGAAAGATGAAGATGTTTCCTTTGAGT
FbMV1_Ep-BL14   AATTCAATCAAATTGTTAAAGAGTAGATCCGTAAGAAAGATGAAGATGTTTCCTTTGAGT
                ************************************************************

FbMV1_Ep-BL13   AGTTTAACTACTTCTCTTCTTCCTACTTTAGCTGAGGTTAATACCTTAGCTCTAGGTAGA
FbMV1_Ep-N28    AGTTTAACTACTTCTCTTCTTCCTACTTTAGCTGAGGTTAATACCTTAGCTCTAGGTAGA
FbMV1_Ep-BL14   AGTTTAACTACTTCTCTTCTTCCTACTTTAGCTGAGGTTAATACCTTAGCCCTAGGTAGA
                **************************************************.*********
```

```
FbMV1_Ep-BL13   GTGAAGAGAGTTAAAGATAGAATCCGTGTGTCTCACAATTTTTTAGTTTACCTTACTAAG
FbMV1_Ep-N28    GTGAAGAGAGTTAAAGATAGAATCCGTGTGTCTCACAATTTTTTAGTTTACCTTACTAAG
FbMV1_Ep-BL14   GTGAAGAGAGTTAAAGATAGAATCCGTGTGTCTCACAATTTTTTAGTTTACCTTACTAGG
                **********************************************************.*

FbMV1_Ep-BL13   ATGTATCACAATCATGGGAGTGAATTTACCATTAAATGGTTAAAATCTTCTCATGTCGCG
FbMV1_Ep-N28    ATGTATCACAATCATGGGAGTGAATTTACCATTAAATGGTTAAAATCTTCTCATGTTGCG
FbMV1_Ep-BL14   ATGTATCACAATCATGGGAGTGAATTTACCATTAAATGGTTAAAATCTTCTCATGTTGCG
                ********************************************************.***

FbMV1_Ep-BL13   TTACAAAAATTCCTAGGAGGTGATAAACTAAAAAGTTTGAGAGATCTCGAACCGAACTGT
FbMV1_Ep-N28    TTACAAAAATTCTTAGGAGGTGATAAACTAAAAAGTTTGAGAGATCTCGAACCGAACTGT
FbMV1_Ep-BL14   TTACAAAAATACCTAGGAGGTGACAAACTAAAAAGTTTGAGAGATCTCGAACCGAACTGT
                ********** *.**********.************************************

FbMV1_Ep-BL13   CCACTCCCTCGTCTGATTAACGGTTGTCCTGCTTATATTAATAAGAGGGACCGTTCTCTG
FbMV1_Ep-N28    CCGCTCCCTCGTCTGATCAACGGTTGTCCTGCTTATATTAATAAGAGGGACCGTTCTCTG
FbMV1_Ep-BL14   CCGCTCCCTCGTCTGATCAACGGTTGTCCTGCTTATATTAATAAGAGGGACCGTTCTCTG
                **.**************.******************************************

FbMV1_Ep-BL13   ATGAGAGAGGGTAATTCCTTTGTGAGCAGATATTGATTGACTCAATTTGGTATTTATCGA
FbMV1_Ep-N28    ATGAGAGAGGGTAATTCCTTCGTGAGCAGATATTGATTGACTCAATTTGGTATTTATCGA
FbMV1_Ep-BL14   ATGAGAGAGGGTAATTCCTTCGTGAGTAGATACTGATTGACTCAATTTGGTATTTATCGA
                ********************.*****.*****.***************************

FbMV1_Ep-BL13   GTCCTTTTAGGACCTTTTAAACCCAAATTATCAACCATTACTGACTCATATAAGGGTTCA
FbMV1_Ep-N28    GTCCTTTTAGGACCTTTTAAACCCAAATTGTCAACCATTACTGACTCATATAAGGGTTCA
FbMV1_Ep-BL14   GTCCTTTTAGGACCTTTTAAACCCAAATTATCAACCATTACCGACTCATATAAGGGTTCA
                *****************************.***********.******************

FbMV1_Ep-BL13   GTTCGAGCATCTCTTGAACTAATTCCTTTGGCTTTTAATCCTTTCATTAACTTTTACGAT
FbMV1_Ep-N28    GTTCGAGCATCTCTTGAACTAATTCCTTTGGCCTTTAATCCTTTCATTAACTTTTACGAT
FbMV1_Ep-BL14   GTTCGAGCATCTCTTGAACTAATTCCTTTGGCCTTTAATCCTTTCATTAACTTTTACGAT
                ********************************.***************************

FbMV1_Ep-BL13   AAGATAGATTTTAGAGCCCCTACTACTCTGTTTTTGAGTCATAAGGGTTCTCCATCTAAT
FbMV1_Ep-N28    AAGATAGATTTTAGAGCCCCTACTACTCTATTTTTGAGTCATAAGGGTTCTCCATCTAAT
FbMV1_Ep-BL14   AAGATAGATTTTAGAGCCCCTACTACTCTGTTTTTGAGTCATAAGGGTTCACCATCTAAT
                *****************************.******************** *********
```

```
FbMV1_Ep-BL13   TCTTTATCGTACAAAGGTATTTTAACAGACTTTTGTCTCTTGAAGTATTCTAGTGTTAGA
FbMV1_Ep-N28    TCCTTATCGTACAAAGGTATTTTAACAGACTTTTGTCTCTTGAAGTATTCTAGTGTTAGA
FbMV1_Ep-BL14   TCCTTATCGTACAAAGGTATTTTAACAGACTTTTGTCTCTTGAAGTATTCTAGTGTTAGA
                **.*********************************************************

FbMV1_Ep-BL13   TTGTCTGAGATCGATTTTTCTCGACCTAAGCATTCTCCCATCTATGATAACTTATTAAAA
FbMV1_Ep-N28    TTGTCTAAGATCGATTTTTCTCGACCTAAGCATTCTCCCATCTATGATAACTTATTAAAA
FbMV1_Ep-BL14   TTGTCTAAGATCGATTTTTCTCGACCTAAGCATTCTCCCATCTATGATAACTTATTAAAA
                ******.*****************************************************

FbMV1_Ep-BL13   TATCTTGATTTGTTAGAGGTTGAAGGTCGTTTGCCCTTGAGAAGATTCAGATCAGTTCTA
FbMV1_Ep-N28    TATCTTGATTTGTTAGAGGTTGAAGGTCGTTTGCCCTTGAGAAGATTCAGATCAGTTCTA
FbMV1_Ep-BL14   TATCTTGATTTGTTAGAGGTTGAAGGTCGTTTGCCCTTGAGAAGATTCAGATCAGTTCTG
                ***********************************************************.

FbMV1_Ep-BL13   TCTTCACTTGACACTCTAGCTTCTTCGTTGAAGTCAGAGGGTCTTAGTTTAAATACAAAA
FbMV1_Ep-N28    TCTTCACTTGACACTCTAGCTTCTTCATTGAAGTCAGAGGGTCTTAGTTTAAATACAAAA
FbMV1_Ep-BL14   TCCTCACTTGACACTCTAGCTTCTTCGTTGAAGTCGGAGGGTCTTAGTTTAAATACAAAA
                **.***********************.********.************************

FbMV1_Ep-BL13   GTGAGTATAAGAAATGGTCTGTCTCAGTTCGCCGTTAAAGAGGAGGCTGCTGGAAAATTA
FbMV1_Ep-N28    GTGAGTATAAGAAATGGTCTGTCTCAGTTCGCTGTTAAAGAGGAGGCTGCTGGAAAATTA
FbMV1_Ep-BL14   GTGAGCATGAGAAATGGTCTGTCTCAGTTCGCCGTTAAAGAGGAGGCTGCTGGAAAATTA
                *****.**.***********************.***************************

FbMV1_Ep-BL13   AGAGTTTTTGCTCTTTTAGATTCCATAAGTCAGTCTGCTCTTAAACCGCTTCATGAGTTT
FbMV1_Ep-N28    AGAGTTTTTGCTCTTTTAGATTCCATAAGTCAGTCTGCTCTTAAACCGCTTCATGAGTTT
FbMV1_Ep-BL14   AGAGTTTTTGCTCTTTTAGATTCCATAAGTCAGTCTGCTCTGAAACCGCTTCATGAGTTT
                ***************************************** ******************

FbMV1_Ep-BL13   TTATTTGACGTCCTGCGTCGGTTACCTAATGATGGTACATTCGACCAGGATGCCTCAGTT
FbMV1_Ep-N28    TTATTTGATGTCCTGCGTCGGTTACCTAATGATGGTACATTCGACCAGGATGCCTCAGTT
FbMV1_Ep-BL14   TTATTTGATATCCTGCGTCGGTTACCTAATGATGGTACATTCGACCAGGATGCCTCAGTT
                ********..**************************************************

FbMV1_Ep-BL13   AAGAGGTCAAATGAAAAATTTTCTCAGYTTGGTATCGCATATTCTTTTGACCTATCTGCT
FbMV1_Ep-N28    AAGAGATCAAATGAAAAATTTTCTCAATTTGGTATCGCATATTCTTTTGACCTATCTGCT
FbMV1_Ep-BL14   AAGAGATCAAATGAAAAATTTTCTCAATTTGGTATCGCATATTCTTTTGACCTATCTGCT
                *****.********************. ********************************
```

```
FbMV1_Ep-BL13   GCTACTGATAGATTACCTGTAGATCTTACAGGTTCTATATTAGAGCAAATGGTAGGTATT
FbMV1_Ep-N28    GCTACTGATAGATTACCTGTAGATCTTACAGGTTCTATATTAGAGCAAATGGTAGGTATT
FbMV1_Ep-BL14   GCTACTGATAGATTACCTGTAGATCTTACAGGTTCTATATTAGAGCAACTGGTAGGTATT
                ************************************************ ***********

FbMV1_Ep-BL13   AAGGGTTTCTCTTCTTCTTGAAAGTCGATTATGGTTGACCGGGATTTTCAATTCCCAGAA
FbMV1_Ep-N28    AAGGGTTTCTCTTCTTCTTGAAAGTCGATTATGGTTGACCGGGATTTTCAATTCCCAGAA
FbMV1_Ep-BL14   AAGGGTTTCTCTTCTTCTTGAAAGTCGATTATGGTTGACCGGGATTTTCAATTCCCAGAA
                ************************************************************

FbMV1_Ep-BL13   GCCGTCGTCGATAAATATTTCAAGGGAGAGGACCCCGGATATATCCGATACTCCGTAGGG
FbMV1_Ep-N28    GCCGTTGTCGATAAATATTTCAAGGGAGAGGACCCCGGATATATTCGATACTCCGTAGGG
FbMV1_Ep-BL14   GCCGTTGTCGATAAATATTTCAAGGGAGAGGACCCCGGATATATTCGATACTCCGTAGGG
                *****.**************************************.***************

FbMV1_Ep-BL13   CAACCAATGGGTGGTCTTAGTTCTTGAGCAGGACTTGCTATCACTCATCACTGAATTCTC
FbMV1_Ep-N28    CAACCAATGGGTGGTCTTAGTTCTTGAGCAGGACTTGCTATCACTCATCACTGAATTCTC
FbMV1_Ep-BL14   CAACCAATGGGTGGTCTTAGTTCTTGAGCAGGACTTGCTATCACTCATCACTGAATTCTC
                ************************************************************

FbMV1_Ep-BL13   CAACTATGTTCATCTCGTCTTAATAATTTAGGAtgaGAGACTCGTTATGAGATTCTCGGT
FbMV1_Ep-N28    CAACTATGTTCATCTCGTCTTAATAATTTAGGATGAGAGACTCGTTATGAGATTCTCGGT
FbMV1_Ep-BL14   CAACTATGTTCTTCTCGTCTTAATAATTTAGGATGAGAGACTCGTTATGAGATTCTCGGT
                *********** ************************************************

FbMV1_Ep-BL13   GATGATATAGTTATCTTTGACTCAAAGTTAGCGAAGATGTACCTTTATGTTATGCAAGAG
FbMV1_Ep-N28    GATGATATAGTTATCTTTGACTCAAAGTTAGCGAAGATGTACCTTTACGTTATGCAAGAG
FbMV1_Ep-BL14   GATGATATAGTTATCTTTGACTCAAAGTTAGCGAAGATGTACCTTTATGTTATGCAAGAG
                ***********************************************.************

FbMV1_Ep-BL13   TTGGGTCTCGAGGTTAATTCCTCGAAATCAATTAATGCTTCTCATTYAGGTTTCGAATTC
FbMV1_Ep-N28    TTGGGTCTCGAGGTTAATTCCTCGAAATCAATTAATGCATCTCATTCAGGTTTCGAATTC
FbMV1_Ep-BL14   TTGGGTCTCGAGGTTAATTCCTCGAAATCAATTAATGCTTCTCATTCAGGTTTCGAATTC
                ************************************** ******* *************

FbMV1_Ep-BL13   GCGAAACGAACTATGATCAATGGGGTAGACGTTTCTGCCCTTTCTCTTCAGCAGATGTTG
FbMV1_Ep-N28    GCGAAACGAACTATGATCAATGGGGTAGACGTTTCTGCCCTTTCTCTTCAGCAGATGTTG
FbMV1_Ep-BL14   GCGAAACGAACTATGATCAATGGGGTAGACGTTTCTGCCCTTTCTCTTCAGCAGATGTTG
                ************************************************************
```

```
FbMV1_Ep-BL13   AGTTCAACTTCTTTAGGTTCTAAAGTTATTGACACTTTCGTCTATTGTCGAAAAGGGTTA
FbMV1_Ep-N28    AGTTCAACTTCTTTAGGTTCTAAAGTTATTGATACTTTCGTCTATTGTCGAAAAGGGTTA
FbMV1_Ep-BL14   AGTTCAACTTCTTTAGGTTCTAAAGTTATTGACACTTTCGTCTATTGTCGAAAAGGGTTA
                ********************************.***************************

FbMV1_Ep-BL13   GTTTCCTCTATATCTCATTTTTCCCGTCTTCTGACTGGTATCCCTGATAACTCAGTTTTT
FbMV1_Ep-N28    GTTTCCTCTATATCTCATTTTTCCCGTCTTCTGACTGGTATCCCTGATAACTCAGTTTTT
FbMV1_Ep-BL14   GTTTCCTCTATATCTCATTTTTCCCGTCTTCTGACTGGTATCCCTGATAACTCAGTTTTT
                ************************************************************

FbMV1_Ep-BL13   AGAAGAATTTCTAAAACTGGGTTACCAGCGTTATCCTTTCTTAATGTACTGTGATCAGCT
FbMV1_Ep-N28    AGAAGAATTTCTAAAACTGGGTTACCAGCGTTATCCTTTCTTAATGTACTGTGATCAGCT
FbMV1_Ep-BL14   AGAAGAATTTCTAAAACTGGGTTACCAGCGTTATCCTTTCTTAATGTACTGTGATCAGCT
                ************************************************************

FbMV1_Ep-BL13   GATCTGGTACAGTTGAGAATTGTTTTAGAGGCAGTAGTCAATCCTAAATATGAGGATTTT
FbMV1_Ep-N28    GATCTGGTACAGTTGAGAATTGTTTTAGAGGCAGTAGTCAATCCTAAATATGAGGATTTT
FbMV1_Ep-BL14   GATCTGGTACAGTTGAGAATTGTTTTAGAGGCAGTAGTCAATCCTAAATATGAGGATTTT
                ************************************************************

FbMV1_Ep-BL13   GACTTCgATAGGTCGAAATTCGACCTACCACTGGTCTCTTTACTAGGATATATGCTGGAC
FbMV1_Ep-N28    GACTTCGATAGGTCGAAATTCGACTTACCACTGGTCTCTTTACTAGGATATATGCTGGAC
FbMV1_Ep-BL14   GACTTCGATAGGTCGAAATTCGACCTACCACTGGTCTCTTTACTAGGATATATGCTGGAC
                ************************.***********************************

FbMV1_Ep-BL13   ATTGCAAATGGTTCAGTCGATAAGACTTACCCATTTTCAATGGAATCCGATAGGCGTGAC
FbMV1_Ep-N28    ATTGCAAATGGTTCGGTCGATAAGACTTACCCATTTTCAATGGAATCCGATAGGCGTGAC
FbMV1_Ep-BL14   ATTGCAAATGGTTCAGTCGATAAGACTTACCCATTTTCAATGGAATCCGACAGGCGTGAC
                **************.***********************************.*********

FbMV1_Ep-BL13   TTTTCAAGAGATTATGAGCCGCATTTAGCAGCTGTAATCCTTCAGGAATCGTTATCTAAG
FbMV1_Ep-N28    TTTTCAAGAGATTATGAGCCGCATTTAGCAGCTGTAATCCTTCAGGAATCGTTATCTAAG
FbMV1_Ep-BL14   TTTTCAAGAGATTATGAGCCGCATTTGGCAGCTGTAATCCTTCAGGAATCGTTATCTAAG
                **************************.*********************************

FbMV1_Ep-BL13   ATAAAAACTTTATCGAGAGATTACGATCACCTGATTAAGACTGGAGCTTTAGCTTTATTC
FbMV1_Ep-N28    ATAAAAACTTTATCGAGAGATTACGATCACCTGATTAAGACTGGAGCTTTAGCTTTATTT
FbMV1_Ep-BL14   ATAAAAACTTTATCGAGAGATTACGATCACCTGATTAAGACTGGAGCTTTAGCTTTATTC
                ***********************************************************.
```

```
FbMV1_Ep-BL13   AAAGGTAAAGCTTCACCCTTATTGAATAGTCAGTTTGAAGGTTTCTTTTCCGATATCATT
FbMV1_Ep-N28    AAAGGTAAAGCTTCAGCCTTATTGAATAGTCAGTTTGAAGGTTTCTTTTCCGATATCATT
FbMV1_Ep-BL14   AAAGGTAAAGCTTCAGCCTTATTGAATAGTCAGTTTGAAGGTTTCTTTTCCGATATCATT
                *************** ********************************************

FbMV1_Ep-BL13   ATAGATTTAACAAAATTAGATCCATATGATAAGGTTGACGAGATAGAATCTATTCTATAC
FbMV1_Ep-N28    ATAGATTTAACAAAATTAGATCCATATGATAAGGTTGACGAGATAGAATCTATTCTATAT
FbMV1_Ep-BL14   ATAGATTTAACAAAATTAGATCCATATGATAAGGTTGACGAGATAGAATCTATTCTATAC
                ***********************************************************.

FbMV1_Ep-BL13   CGTCATGCTAAGACTCCTTCATGCTCTGTCGGACAGGCTTTAACAATTTTGGAGGAAGTG
FbMV1_Ep-N28    CGTCATGCTAAGACTCCTTCATGCTCTGTCGGACAGGCTTTAACAATTTTGGAGGAAGTG
FbMV1_Ep-BL14   CGTCATGCTAAGACTCCTTCATGCTCTGTCGGACAGGCTTTAACAATTTTGGAGGAGGTG
                ********************************************************.***

FbMV1_Ep-BL13   AATTCTCTTGTCTTTAAATTTACATTTAAGACAGAGATTTCTCGTGTAAAATACGAGAAG
FbMV1_Ep-N28    AATTCTCTTGTCTTTAAATTTACATTTAAGACAGAGATTTCTCGTGTAAAATACGAGAAG
FbMV1_Ep-BL14   AATTCTCTTGTCTTTAAATTTACATTTAAAACAGAGATTTCTCGTGTAAAATACGAGAAG
                *****************************.******************************

FbMV1_Ep-BL13   GATTCATCTCCTATCCTTCAATTGTTAAGAAAGTCAGAAGGGCGCATTCCCATTAACTAT
FbMV1_Ep-N28    GATTCATCTCCTATTCTTCAATTGTTAAGAAAGTCAGAAGGGCGCATTCCCATTAACTAT
FbMV1_Ep-BL14   GATTCATCTCCTATTCTTCAATTGTTAAGAAAGTCAGAAGGGCGCATTCCCATTAACTAT
                **************.*********************************************

FbMV1_Ep-BL13   TGACAGACACCGGTTCTATCTTACTAAccaaggttattggtcttcagattgctttctaca
FbMV1_Ep-N28    TGACAGACACCGGTTCTATCTTACTAAccaaggttattggtcttcagattgctttctgca
FbMV1_Ep-BL14   TGACAGACACCGGTTCTATCTTACTAAccaaggttattggtcttcagattgctttctaca
                *********************************************************.**

FbMV1_Ep-BL13   tcaatttattgatgagacggtatctgctcaaaagggtctcgaagataggattaatcatcc
FbMV1_Ep-N28    tcaatttattgatgagacggtatctgctcaaaagggtctcgaagataggattaatcatcc
FbMV1_Ep-BL14   tcaacttgttgatgagacggtatctgctcaaaagggtctcgaagataggattaatcatcc
                ****.**.****************************************************

FbMV1_Ep-BL13   tggcagcgagggttcccttcgttttcattaacgagaatgcccc
FbMV1_Ep-N28    tggcagcgagggttcccttcgttttcattaacgagaatgcccc
FbMV1_Ep-BL14   tggcagcgagggttcccttcgttttcattaacgagaatgcccc
                *******************************************
```
