# Supplementary material for: Novel Mitoviruses and a Unique Tymo-Like Virus in Hypovirulent and Virulent Strains of the Fusarium Head Blight Fungus, Fusarium boothii
Source: Viruses. 2018 Oct 26;10(11):584. doi: 10.3390/v10110584 (PMC6266667; doi:10.3390/v10110584)
Supplement: Supplementary file 1 [file viruses-10-00584-s001.zip › sup materials/Mizutani_Fig-S3.pdf]

CLUSTAL format alignment by MAFFT (v7.390)

```
AROM_domain     NFPDEEFKTLRELYD---IALKNG--VEFLDL---ELTLPTDIQY-EVINKRGNTKIIGS
FbMV1_AROM      SYKGSV-RASTETIP-----LAFNPFINFYD--KMDFRAPTTTFLSHKGSPSNSLSYKGI
TcMV_AROM       KFTGDR-DGLRALMR---FALKFNPFVEFNKL-DKKSLAPTKFIMSRSASPSNKVSWFGI
BcMV3_AROM      PFKGSI-NEFNDIIQGPVFSIFFDRLNGFPELVKKSNLAPSKVRLLRSSSSSNNVSWHGI
FpMV3_AROM      PYKGSV-DYLNKCLE---ISLNTKWQASIQKICQNHNLAPTTFHMSGKASPSNVNSSTGI
SlaMV5_AROM     PFSGSE-LFLNDLIS---LSLKTPWPKNLLEIASKHNLAPTSFHFSGKASPSNVNSSQGL
                 : ..               :       : .        *:        .  .  .  *

AROM_domain     HHDFQGL-------------YSWDDAEWENRFNQALTLDVDVVKFVGTAVNFEDNL--RL
FbMV1_AROM      LTDFCTLKYSSVRLSEIDFSRPKHSPIYDNLLKYTDLLEV-EGRLP---------L-RRF
TcMV_AROM       LTDTRLL-------------REGSPRLWENIQAYLTMVGADQFRFD---LDYACSLADRL
BcMV3_AROM      ITDSWNI--------------SNSKEMSSNVSNYLASIKA---------------LGWNT
FpMV3_AROM      LSDIYYL-----------LAHPDGAFVFSNILKHLEIVGS---------------V-WNT
SlaMV5_AROM     LSDIYLL-----------LSHPEGDIVYYNLLNYLDNIGV---------------V-WNT
                  *   :                      *       :                 :  .

AROM_domain     EHFRDTHKNKPLIAVNMTSKGSISRVLNNVLTPVTSDLLPNSAAPGQLTVAQINKMYTSM
FbMV1_AROM      RSVTSSTD----------TTASSLK--SEGTSLNTKVSMRNG-------TSQFAVKEEAA
TcMV_AROM       KSFDDKVDSQGQW---IRQVSKSGQVLTQVDSMKSKDSIRGHGIGPGLGLSQFALKEEAA
BcMV3_AROM      MWFNSKLN----------EMIELGDRLNQVGSLKTKKSLSGQ-------FGQFSLKEEAA
FpMV3_AROM      QSFLQRLN----------DGAEIVKRM---SSIQTKCSMRNP-------FGQFAIKEEAA
SlaMV5_AROM     KMFLSRLN----------DAKDIILRLPE-GSLPFKKSMVTP-------FGQFAIKKEAA
                  . .  .             .         :   .  :           .*:     :

AROM_domain     GGIEPKELFVVGKPIGHSRSPILHNTGYEIL-GLPHK--FDKFETESAQLVKEKLLDGNK
FbMV1_AROM      GKL---RVFATLDSMSQSATKPTHEFLFDVTRRLPNDGTFD--QDASVKRSNEKFSQXGI
TcMV_AROM       GKI---RLFALMDSITQSVMSPLHDYMFAILRNIPNDGTFD--QEASIARSQEKAVTAGK
BcMV3_AROM      GKL---RIFAIVDSITQSLLSPLHDFMFDLLKKIPNDGTFD--QDLSVKRSQVKSLSSGK
FpMV3_AROM      GKV---RVFALVDSITQSVMKPIHLGLFKVLRHLPNDGTFD--QDASVTRCSEKASEAGK
SlaMV5_AROM     GKI---RVFALVDSITQSVMKPLHLGLFAVLKQLPNDGTFD--QDASVTRCSIKAQQAGK
                * :   .:*.  ..: :*     *   : :   :*:.  **  :  *    . *    .

AROM_domain     NFG----GAAVTIPLKL--DIMQYMDELTDAAKVIGAVNTVIPLGNKKF
FbMV1_AROM      AYSFDTSAATDRLPVDTTGSMLEQMVGIKGFSSSWKSI-----MVDRDF
TcMV_AROM       AFSYDLTAATDRLPVILTAFILSTIVGIRTFGGLWRSI-----LVKRPF
BcMV3_AROM      AFSFDLSAATDRLPVDLTVKILSKIFS-DEFGTSWKQL-----MVNRDF
FpMV3_AROM      AFSFDLSAATDRLPVSLTGNIIESLFKIPGLSQSWQKV-----MVDRNF
SlaMV5_AROM     AFSFDLSAATDRLPVGLTGSIIESLFQITDLSNSWKSV-----MVDREF
                 :.    .*:  :*:     ::. :      .     :     : .: *
```
