# Supplementary figures and images for: Novel Mitoviruses and a Unique Tymo-Like Virus in Hypovirulent and Virulent Strains of the Fusarium Head Blight Fungus, Fusarium boothii
Source: Viruses. 2018 Oct 26;10(11):584. doi: 10.3390/v10110584 (PMC6266667; doi:10.3390/v10110584)

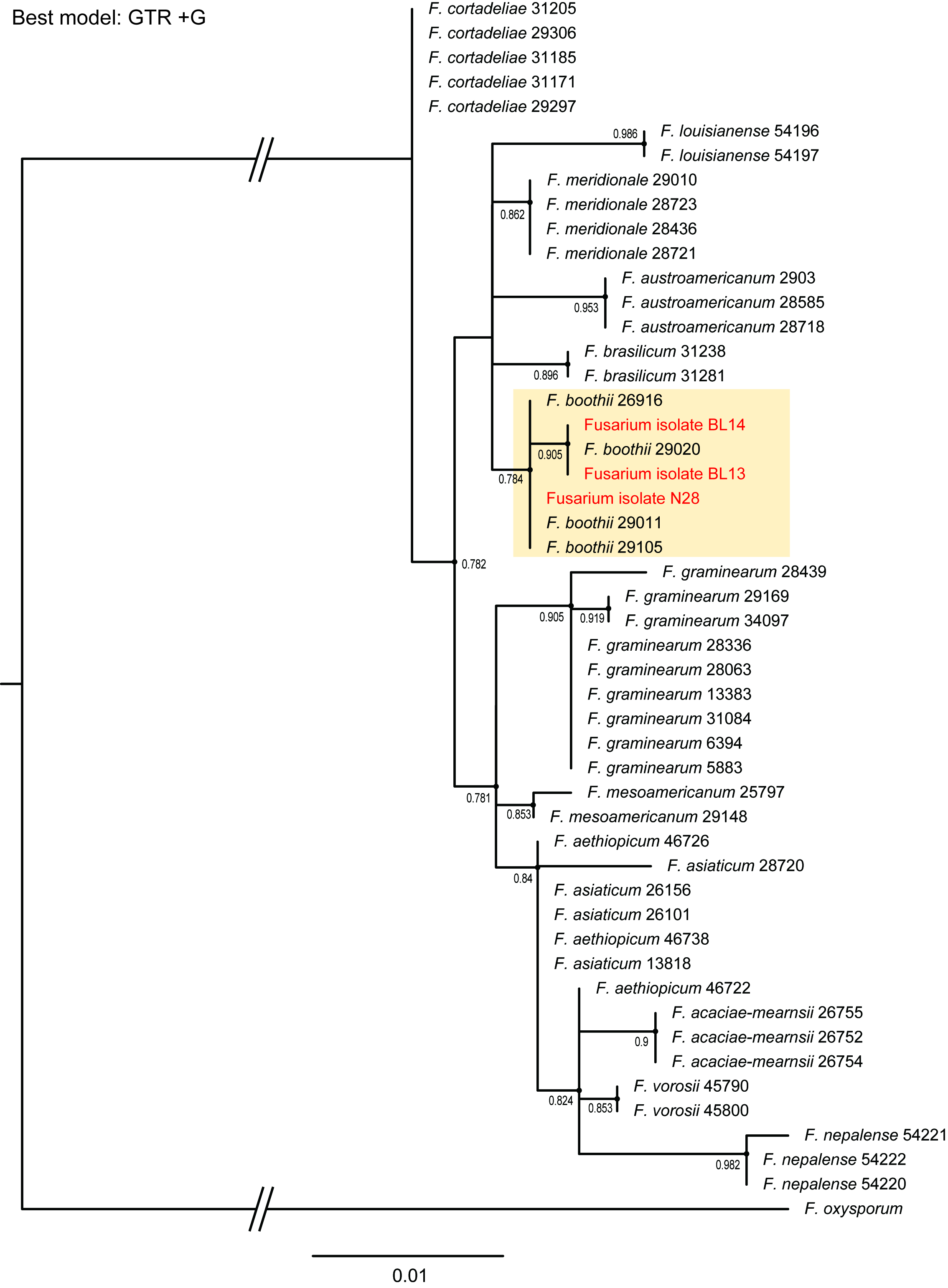

Supplement: Supplementary file 1 [file viruses-10-00584-s001.zip › sup materials/Mizutani_Fig-S1.tif]
